# Persistent HPV infection after conization of cervical intraepithelial neoplasia—— a systematic review and meta-analysis

**DOI:** 10.1186/s12905-023-02360-w

**Published:** 2023-05-03

**Authors:** Yueyang Zhang, Zhiwen Ni, Ting Wei, Qingsong Liu

**Affiliations:** 1 Department of Medical Laboratory, Public Health Clinical Center of Chengdu, Chengdu, China; 2 Department of Radiology, Chengdu First People’s Hospital, Chengdu, China; 3 Department of Medical Laboratory, Nanchong Central Hospital, The Second Clinical Medical College, North Sichuan Medical College, Nanchong, China; 4 Department of Prenatal Diagnosis, Chengdu Women’s and Children’s Central Hospital, School of Medicine, University of Electronic Science and Technology of China, 1617#, Riyue Avenue, Qingyang District, 611731 Chengdu, China

**Keywords:** HPV, Persistent infection, CIN, Conization, Human papillomavirus

## Abstract

**Objective:**

To systematically evaluate several factors of persistent human papillomavirus (HPV) infection following conization in patients with cervical intraepithelial neoplasia (CIN).

**Methods:**

PubMed, EMBASE and the Cochrane Library were searched from January 1, 1998 to September 10, 2021. Random-effects models for meta-analyses were used and pooled relative risks with 95% confidence intervals were reported. Literature screening, data extraction, and assessment of the risk of bias in the included studies were conducted independently by two researchers. Data analysis was performed with Stata software, version 12.0.

**Results:**

A total of 28 studies were included in this study. Meta-analysis revealed that surgical margin and residual disease were positively correlated with persistent HPV infection after conization. Compared with patients infected with other types of HPV, CIN patients with HPV 16 had a higher persistent infection rate (OR = 1.967, 95% CI (1.232–3.140), *P* < 0.05).

**Conclusions:**

CIN patients who are postmenopausal, have positive surgical margins and residual lesions, and are positive for HPV 16 are prone to persistent HPV infection after conization.

**Supplementary Information:**

The online version contains supplementary material available at 10.1186/s12905-023-02360-w.

## Introduction

Human papillomavirus (HPV) is one of the foremost sexually transmitted viruses among young women around the world [1]. Although most HPV infections are transient and cleared in a couple of years after exposure, 10–20% of HPV infections are latent and persistent [2]. Persistent insecure HPV infection is closely and systematically related to high-grade cervical intraepithelial neoplasia (CIN), which is necessary for the progression of cervical precancerous lesions to cervical cancer (CC) [3].

Cervical conization is currently a widely used method for the diagnosis and treatment of CIN [4]. Unfortunately, there is a relatively high incidence of recurrence after conization, especially in patients with high-risk human papillomavirus (HR-HPV) [5, 6]. Moreover, the pooled hazard was higher for patients who underwent a minimally invasive approach than for patients who underwent open radical hysterectomy [7]. Surgical margin, CIN grade, surgical method, endocervical gland involvement (EGI), age, parity and immunological dysfunction are potential factors affecting the persistence and/or recurrence of HPV [8]. Therefore, assessing these factors that contribute to persistent HR HPV infection after cervical conization may help identify women at higher risk of disease recurrence. Here, we performed a systematic review and meta-analysis to explore the implication of personal history (e.g., menopausal, marriage, smoking status) and clinical factors (e.g., surgical margin, endocervical gland involvement, CIN types, etc.) related to persistent HPV infection after conization of CIN.

## Methods

### Search strategy and selection criteria

PubMed, EMBASE, and the Cochrane Library were searched from January 1, 1998 to September 10, 2021. The domains of the search terms were human papillomavirus (HPV), cervical intraepithelial neoplasia (CIN), conization, and loop excision. We combined conization and loop excision with the Boolean operator “OR”, and the result was combined with the other terms with “AND”.

**Fig. 1:**
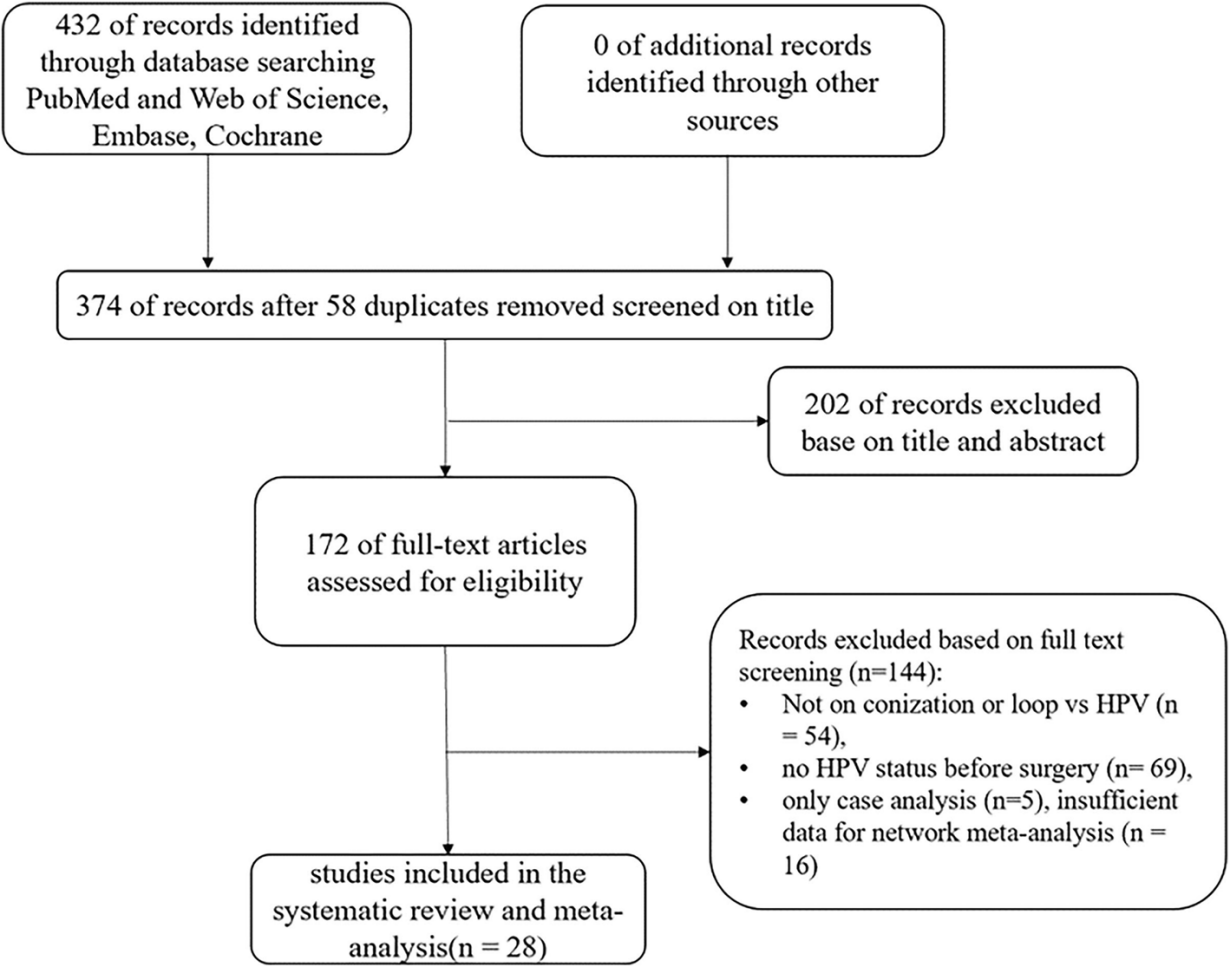
Flowchart of computerized search and the eligible studies included in this systematic review and meta-analysis

It was eligible if the study reported the main results of interest, which was persistent HPV infection after conization of CIN. We excluded those studies that did not mention HPV infection before surgery, if those patients underwent radical hysterectomy or if those articles met any of the following conditions: comments, case reports, reviews, conference records, and communications (Fig. 1).

### Data extraction

All studies were independently reviewed and evaluated critically by 2 researchers. All information was extracted independently in duplicate manner by 2 researchers. Data extraction included study characteristics (author, year, study period) and information on personal history (e.g., patient age, menopausal status, marriage status, smoking status) and clinical factors (e.g., surgical margin, endocervical gland involvement, CIN types, residual disease, cervical invasive carcinoma, type of HPV) related to infection and/or persistent infection of HPV.

### Statistical analyses

Odds ratios with 95% confidence intervals (CIs) were calculated. The inconsistency index (*I*^*2*^) and Q statistics were measured. The heterogeneity between studies was assessed by Cochran Q (*P* < 0.1) and I^2^ (> 50%) tests. The random effects model was used to calculate the pooled effect, and a bilateral *P* value < 0.05 was considered statistically significant. A sensitivity analysis was performed by removing one study at a time from the meta-analysis and then assessing its impact on the combined results. We have a tendency to use Begg’s and Egger’s tests to determine whether there was publication bias. The funnel plot was not used for publication bias because more than ten studies were needed for such analysis. All analyses were carried out using Stata, version 12.0.

### Patient involvement

None because the study was based on published literature.

## Results

### Study selection

A total of 202 papers were deleted during the initial title and abstract screening among the 432 retrieved papers. Another 144 papers were excluded after being reviewed fully. A total of 28 papers were finally enrolled in the systematic review and meta-analysis.

The features of the 28 papers are illustrated in Table 1. The number of patients ranged from 31 to 1734 in the 28 selected papers. The recruitment of patients came from two types of research, 12 prospective studies, and 16 retrospective studies.

**Table 1:** The basic characteristics of the included studies

Author（year）	Study type	Study size	Study setting	Consecutive recruitmen	Age	Study period	Country	Blinding
Huei-Jean Huang 2021 [9]	prospective	493	Hospital	N/A	Mean:40.9 range (20.2- 78.0)	2008-2014	China	NO
Yung-Taek Ouh 2020 [10]	retrospective	1029	Hospital	N/A	Mean:41.96±12.63	2014 - 2018	Korea	NO
M-E Fernandez-Montol 2020 [11]	retrospective	242	Hospital	N/A	Mean:37.4±10.9	2006-2016	Gynecology	NO
Aiping Fan 2018 [12]	retrospective	172	Hospital	N/A	Mean:39.1 range (23-70)	2006-2016	China	NO
Derya Kilic 2020 [13]	retrospective	395	Hospital	N/A	Mean:42.66±8.97	N/A	Turkey	NO
Anna So¨derlund-Strand 2014 [14]	prospective	178	Hospital	N/A	N/A	2001-2003	Sweden	NO
Rosario Lara-Peñaranda 2020 [15]	retrospective	265	Hospital	N/A	Mean:36.53±9.53	2011-2016	Spain	NO
Jung Mi Byun 2018 [16]	prospective	172	Hospital	Yes	Mean:39.4±10.7	2010 - 2014	Korea	NO
Kristin Friebe 2017 [17]	Prospective	144	Hospital	N/A	Mean:36.1 range( 21.8-68.5)	2007-2013	Germany	NO
Young-Tak Kim 2010 [18]	prospective	287	Hospital	N/A	Mean:40.7 range(19-67)	2000-2007	Korea	NO
Luca Giannella 2017 [19]	retrospective	298	Hospital	N/A	N/A	2012-2014	Italy	NO
Theresa Maria Kolben 2019 [20]	prospective	100	Hospital and German study centerst	N/A	Mean:31.6 range(23.8-47.3) and Mean:31.0 range(23.9-43.4)	2014-2016	Germany	YES
Immaculada Alonso 2006 [21]	retrospective	224	Hospital	N/A	Mean38.6 range( 22-83)	1998-2004	Barcelona	NO
Kyeong A So 2018 [22]	retrospective	160	Cheil General Hospital & Women’s Healthcare Center	N/A	Mean:38.1 range(18–86)	2014	Korea	NO
Kouichiro Kawano 2021 [23]	retrospective	439	Hospital	N/A	Mean:35 range(20–78)	2007-2016	Japan	NO
Jeong-Yeol Park 2009 [24]	prospective	115	Hospital	N/A	Mean:47 range(26–73) vs Mean:47 range(31–75)	2007-2008	Korea	NO
Lin Jing 2018 [25]	retrospective	594	Hospital	N/A	Mean:45 range(40-51) vs Mean:47 range(42.56)	2006-2015	China	NO
O.K. Vintermyr 2014 [26]	retrospective	58	Hospital	N/A	Mean:37.6 ±5.9	1998-2003	Norway	NO
A.L. Diste´fano 1998 [27]	prospective	36	Hospital	N/A	Mean:31 range (17 - 45)	N/A	Argentina	NO
Iztok Takaˇc 2008 [28]	prospective	797	Hospital	N/A	Mean: 39.9 range(29-70)	1999-2004	Slovenia	NO
J. E. Palmer 2016 [29]	retrospective	2093	Hospital	N/A	Mean:30 range(18–68)	2007-2012	UK	NO
Eralp Baser 2014 [8]	retrospective	292	Hospital	N/A	Mean:39.8 range(20–71)	2007-2012	Turkey	NO
Akihiro Karube 2021	prospective	169	Hospital	N/A	range 20–49	2008-2018	Japan	NO
Jeong-Yeol Park 2008 [30]	retrospective	236	Hospital	N/A	Mean:40 range(25–72) vs Mean:41 range(23–75)	2001-2016	Korea	NO
Lu´ıs Otávio Zanatta Sarian 2004 [31]	prospective	94	Hospital	N/A	Mean:34 range(20-60)	2001-2002	Brazil	NO
Akiko Kudoh 2016 [32]	retrospective	211	Hospital	N/A	Mean:34 range(20–72) vs Mean:37 range(18–79)	2009-2013	Japan	NO
SONIA ANDERSSON 2021 [33]	prospective	529	Hospital	N/A	Mean:34.3	2014-2017	Sweden	NO
Spinillo Arsenio 2020 [34]	retrospective	2985	Hospital	N/A	Mean:36.8 ± 10.4 range(21-65)	2010-2018	Italy	NO

### The personal history of patients with persistent HPV infection

**Fig. 2:**
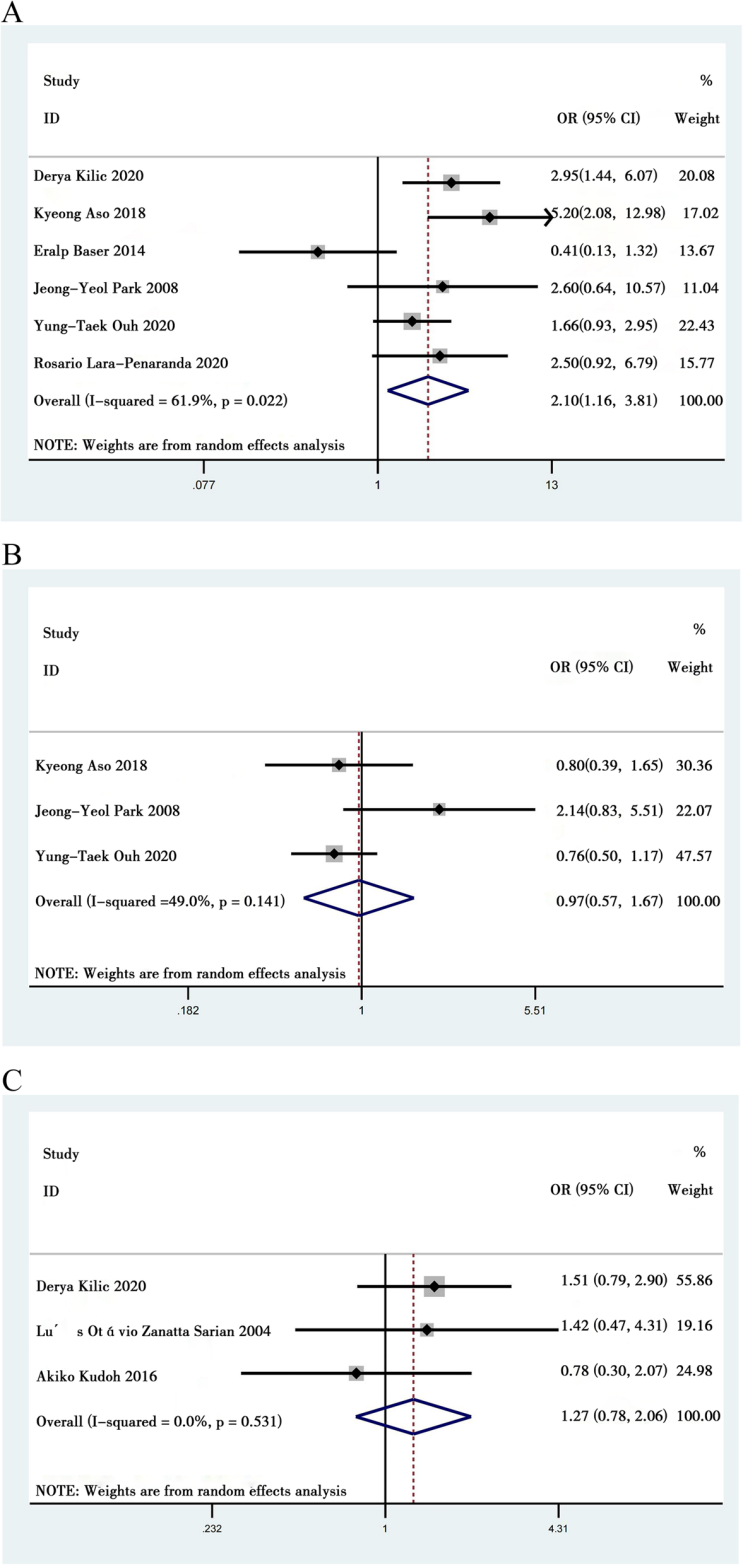
Meta analysis of the personal history of patients associated with persistent HPV infection. **A** Menopausal status correlated with persistent HPV infection. **B** Marriage status correlated with persistent HPV infection. **C** Smoking correlated with persistent HPV infection

Yung-TaekOuh et al. [10], Derya Kilic et al. [13], Rosario Lara-Peñaranda et al. [15], Kyeong A So et al. [22], Eralp Baser et al. [8], and Jeong-Yeol Park et al. [30] reported the menopausal status in CIN patients after surgery with HPV persistence compared to without HPV persistence. Moderate heterogeneity was found in the six articles (*I*^*2*^ = 61.9%). A random-effect model was adopted during this analysis. The comprehensive analysis found that persistent HPV infection during conization was influenced by menopausal status. Postmenopausal status was positively correlated with persistent HR-HPV infection (OR = 2.098, 95% CI: 1.156 to 3.809, *P* = 0.015) (Fig. 2A).

Yung-Taek Ouh et al. [10], Kyeong A So et al. [22], and Jeong-Yeol Park et al. [30] reported on the marital status in CIN patients after surgery with HPV persistence compared to those without HPV persistence. Low heterogeneity was found in the 3 studies (*I*^*2*^ = 49%). The comprehensive analysis found that persistent HPV infection after conization or LEEP was not obviously correlated with marital status (OR = 0.972, 95% CI: 0.566 to 1.670, *P* = 0.919) (Fig. 2B).

We identified 3 eligible studies [13, 31, 32] including 431 CIN patients after surgery to investigate smoking correlated with persistent HPV positivity. Our meta-analysis showed that smoking had no effect on persistent HPV positivity (OR = 1.268; 95% CI: 0.78 to 2.062, *P* = 0.339, Fig. 2C). Low heterogeneity was found in the three studies (*I*^*2*^ = 0%).

### The clinical factors associated with persistent HPV infection

**Fig. 3:**
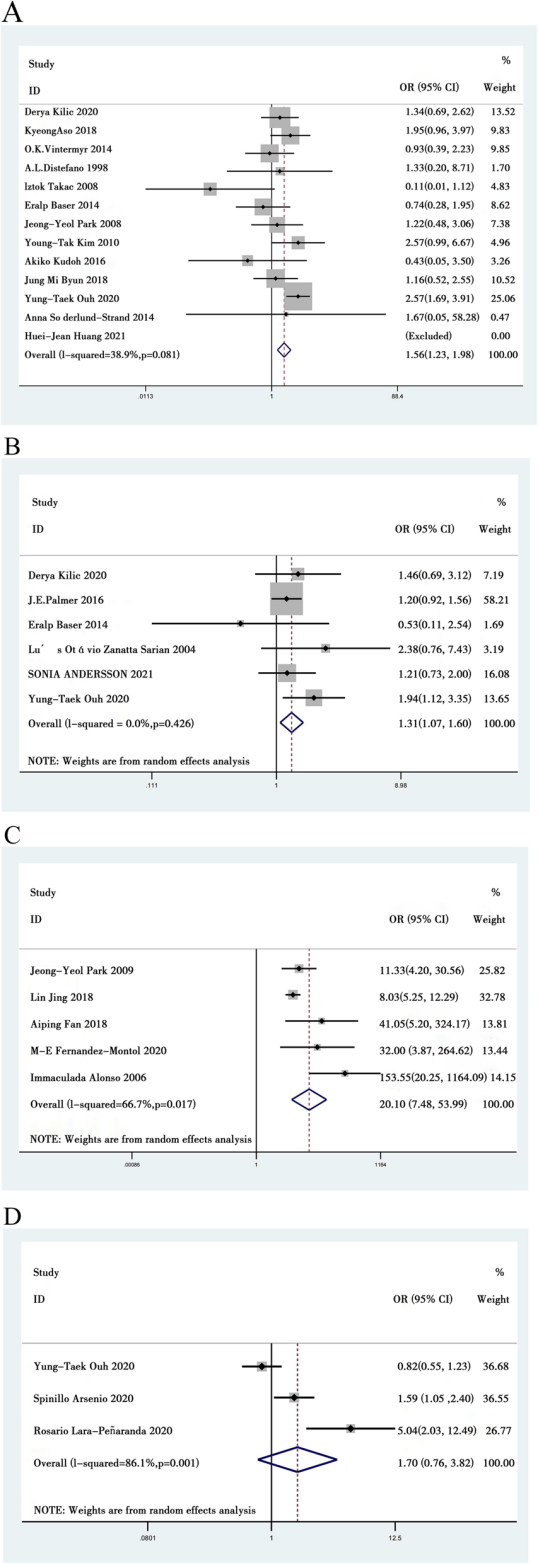
Meta analysis of the clinical factors associated with persistent HPV infection. **A** The type of CIN correlated with persistent HPV infection. **B** Surgical margin correlated with persistent HPV infection. **C** Residual disease correlated with persistent HPV infection. **D** EGI correlated with persistent HPV infection

We identified 13 eligible studies [8-10, 13, 14, 16, 18, 22, 26-28, 30, 32] that reported the type of CIN in patients after surgery with HPV persistence compared to without HPV persistence. According to the literature, we divided CIN types into CIN2 and CIN3 for meta-analysis. Our meta-analysis showed that the type of CIN (CIN2 or CIN3) had no effect on persistent HPV infection (OR = 1.383; 95% CI: 0.972 to 1.967, *P* = 0.072, Fig. 3A). Low heterogeneity was found in the 13 studies (*I*^*2*^ = 38.9%).

We identified 6 eligible studies [8, 10, 13, 29, 31, 33] that reported surgical margins in patients after surgery with HPV persistence compared to those without HPV persistence. Our meta-analysis revealed that surgical margin was positively correlated with persistent HPV infection (OR = 1.309; 95% CI: 1.068 to 1.603, *P* = 0.009, Fig. 3B). Moderate heterogeneity was found in the 6 studies (*I*^*2*^ = 0%).

We identified 5 eligible studies [11, 12, 21, 24, 25] that reported residual disease in patients after surgery with persistent HPV infection compared to patients without persistent HPV infection. In those studies, they identified residual lesions through examinations, including liquid-based cytology tests (LCTs), HPV tests, and colposcopy. Our meta-analysis revealed that residual disease was positively correlated with persistent HPV infection (OR = 20.102; 95% CI: 7.485 to 53.987, *P* = 0.000, Fig. 3C). Moderate heterogeneity was found in the five studies (*I*^*2*^ = 66.7%).

We identified 3 eligible studies [10, 15, 34] that reported endocervical gland involvement (EGI) in patients after surgery with HPV persistence compared to those without HPV persistence. EGI means that cervical biopsy involves glands. Our meta-analysis revealed that EGI had no effect on persistent HPV infection (OR=1.700; 95% CI: 0.757 to 3.818, *P* = 0.198, Fig. 3D). High heterogeneity was found in the three studies (*I*^*2*^ =86.1%).

### The type of HPV correlated with persistent HPV infection

**Fig. 4:**
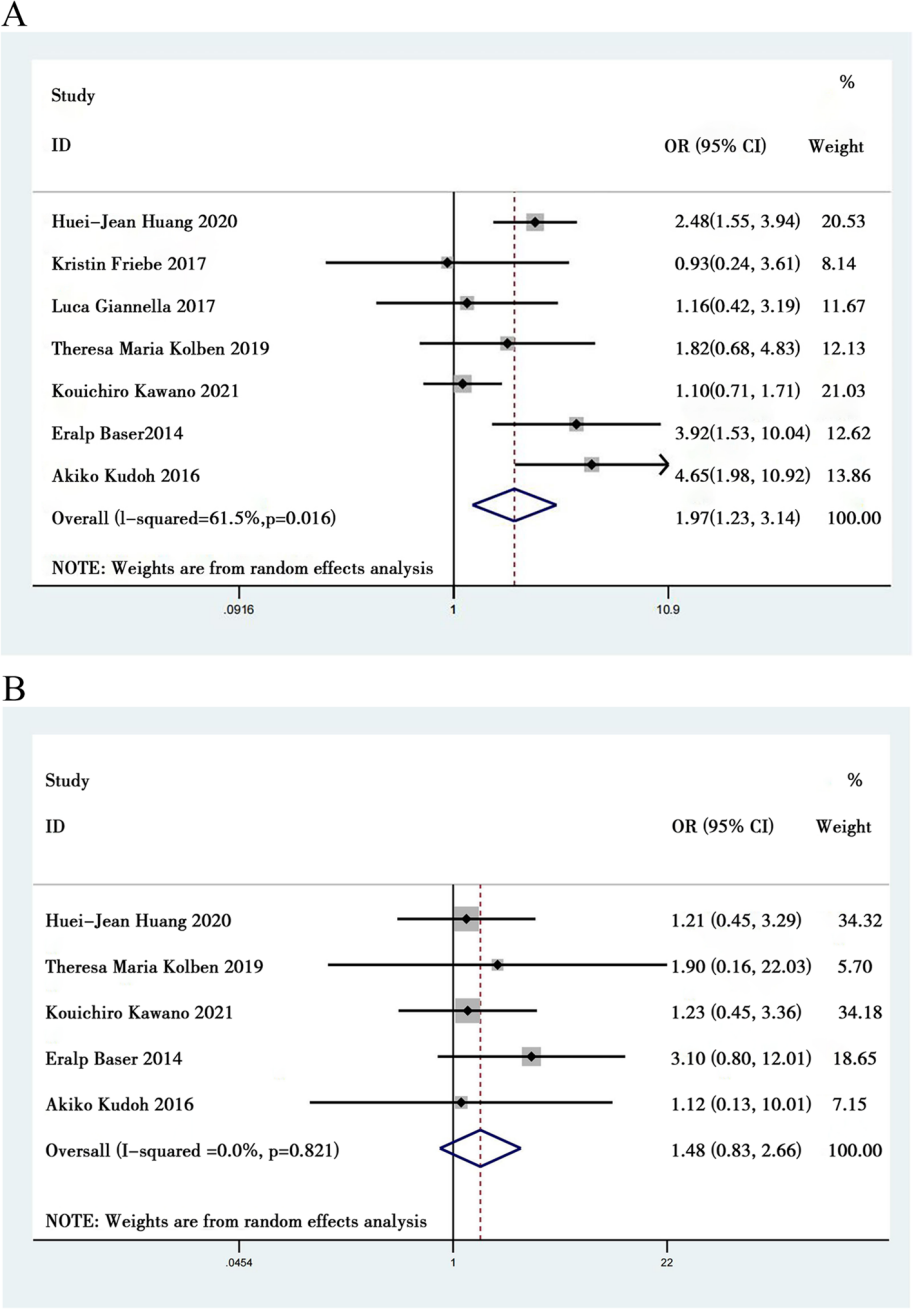
Meta analysis of the type of HPV correlated with persistent HPV infection. **A** HPV16 correlated with persistent HPV infection. **B** HPV18 correlated with persistent HPV infection

We identified 7 eligible studies [8, 9, 17, 19, 20, 23, 32] including 1790 CIN patients after surgery to investigate the relationship between HPV types and persistent HPV infection and nonpersistent HPV infection. Our results revealed that there was a positive effect on persistent HPV infection between HPV 16 and other types of HPV. (OR = 1.967; 95% CI: 1.232 to 3.140, *P* = 0.005, Fig. 4A). Moderate heterogeneity was found in the 7 studies (*I*^*2*^ = 61.5%).

We identified 5 eligible studies [8, 9, 20, 23, 32] including 1516 CIN patients after surgery to investigate the relationship between HPV types and persistent HPV infection and nonpersistent HPV infection. Our results revealed that there was no effect on persistent HPV infection between HPV 18 and other types of HPV. (OR = 1.482; 95% CI: 0.826 to 2.661; *P* = 0.187, Fig. 4B). Low heterogeneity was found in the 5 studies (*I*^*2*^ = 0%).

### Sensitivity analysis

**Fig. 5:**
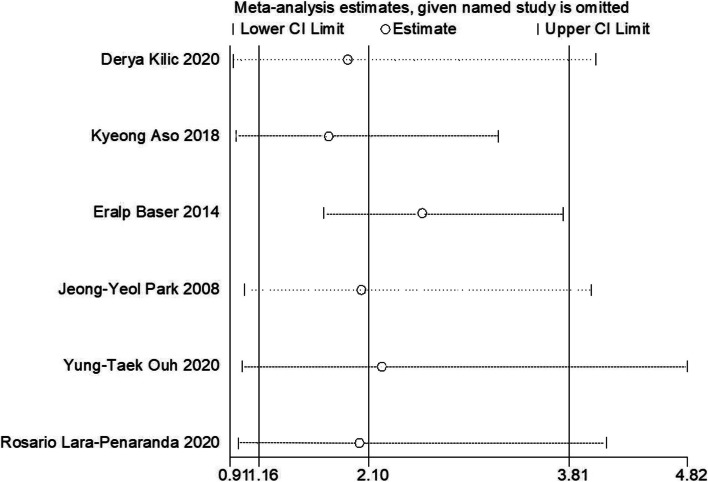
Sensitivity analysis for testing OR for the menopausal status

In our analysis on menopausal status in patients with persistent HPV infection, heterogeneity was discovered in the studies (*I*^*2*^ = 61.9%). Sensitivity analysis was used to eliminate individual studies sequentially. The results indicated that there was one study (the 13^th^ study) that contributed considerably to heterogeneity (Fig. 5). The pooled OR (OR = 2.553, 95% CI: 1.713 to 3.805, *P* = 0.000) of the remaining studies changed the final trend after withdrawing this study. In our sensitivity analysis, the number of patients in the 13^th^ study was less than that in the other studies.

In our analysis of the EGI with persistent HPV infection, heterogeneity was observed in the three studies (*I*^*2*^ = 86.1%). Sensitivity analysis was performed to eliminate individual studies sequentially. The results indicated that no studies made a significant contribution to heterogeneity. Moreover, the pooled OR of the remaining studies did not change the final trend.

### Publication bias

In the meta-analysis on the type of CIN in patients after surgery with persistent HPV infection, 13 eligible studies were identified. No evidence of publication bias was found in our study through funnel plot, Egger’s test (*P* > 0.05) and Begg’s test (*P* > 0.05).

## Discussion

Our research is the first meta-analysis investigating the factors correlated with the role of HPV status after CIN conization. This result indicated that menopausal status, surgical margin, residual disease and HPV 16 correlated positively with persistent HPV infection after conization, while marriage, smoking, type of CIN (CIN2 or CIN3), EGI, and HPV18 had no impact on persistent HPV infection.

Naouel Tifaoui et al. [35] reported that follow-ups of women with normal cytology and positive HPV showed a higher percentage of HPV persistence among menopausal women. HPV infections in young patients are mostly transient, and corresponding symptoms are rarely seen. In contrast, older people are prone to persistent infection and cervical cancer. In postmenopausal women, immunity declines with age, leading to a long time for virus clearance after HPV infection. Here, our study also revealed that menopausal status correlated positively with persistent HPV infection after conization. One study showed that smoking is an important factor that increases the risk of HR-HPV persistence [36]. Smoking is an immunosuppressant and one of the significant factors leading to the development of cervical cancer [37]. However, our study revealed that smoking did not have any impact on persistent HPV infection after conization. Marriage may be related to sexual contact, contraception, childbirth and other factors that may affect HPV reinfection. Our study shows that persistent HPV infection after conization or LEEP was not obviously correlated with marriage. We should refine other aspects about marriage, but there is not enough data.

The surgical margin and residual disease showed the residual status of the cervix after surgery. The residual rate after surgery varies among studies, mainly due to the dearth of standardization of the conization operation and the totally different applied mathematics strategies used for analysis [25]. Nonetheless, our results showed that both surgical margins and residual disease were positively associated with persistent HPV infection. Studies have reported that the resection failure rate defined as persistent or recurrent CIN grade 2 or more severe (CIN2 +) was 4–18%, and most cases were diagnosed 2 years after initial treatment [6, 38, 39]. Therefore, we analyzed the influence of CIN2 and CIN3 in patients with HPV persistence after conization. Unexpectedly, the type of CIN (CIN2 or CIN3) in patients after conization did not have any impact on persistent HPV infection. However, the relationship between the severity of cervical precancerous lesions and the ongoing risk was unclear, and patients with inferior cervical lesions showed higher infectious viral loads than CIN-3 patients [40]. Yung-Taek Ouh et al. [10] found that the HPV persistence rate in CIN-2 patients after treatment was higher than that in CIN-3 patients, which was inconsistent with previous research reports [41]. Our research found that EGI did not have any impact on persistent HPV infection. The standard treatment for CIN, especially high-grade lesions, is conization or LEEP [42]. Even if the lesion was completely resected, patients with high-grade lesions have a higher risk of recurrence than the general population [43]. Therefore, when patients have endocervical gland involvement, they usually choose further surgical treatment, such as a hysterectomy. This may be one reason that EGI did not have any impact on persistent HPV infection after conization.

HPV 16 or HPV 18 had a negative impact on the survival rate in patients with cervical cancer [44]. Our meta-analysis revealed that HPV 16, not HPV 18, has a positive impact on persistent HPV infection compared to other types of HPV after conization. In our included meta-analysis studies, there were a few patients who were diagnosed with HPV 18 before conization. Thus, it was not valuable to HPV 18 persistence infection after conization in our study because the number of patients studied was too small.

We also acknowledge that our meta-analysis has several limitations. First, all the relevant factors analyzed were single factors, and there may be interactions between different factors. Therefore, in the future, it is expected that a larger sample size test will be used for further verification and correction. Second, the Laparoscopic Approach to Cervical Cancer (LACC) trial questioned the safety of minimally invasive surgery [45], and our study discussed persistent HPV infection after conization. Of course, in addition to conization, there are other treatments. Additionally, due to the inherent limitations of retrospective studies, more large-scale irregular clinical trials are required to verify our results. Last but not least, these studies recruited a wide range of patients, from CIN I to CIN III and even carcinoma in situ, which may increase the heterogeneity of the meta-analysis. A review showed that the surgical treatment of CIN was associated with an increased risk of obsterics [46], probably due to persistent HPV infection after surgery. Nevertheless, our study lays the foundation for further research in understanding the factors influencing persistent HPV infection after conization.

## Conclusion

CIN patients with postmenopausal status, positive surgical margin and residual lesions, and HPV 16 are more likely to have persistent HPV infection after conization.

## Supplementary Information

**Additional file 1.**

## Data Availability

All data generated or analysed during this study are included in these published articles [and their supplementary information files]. 1) Role of human papillomavirus status after conization for high‐grade cervical intraepithelial neoplasia. 2) Risk factors for type-specific persistence of high-risk human papillomavirus and residual/recurrent cervical intraepithelial neoplasia after surgical treatment. 3) Long‐term predictors of residual or recurrent cervical intraepithelial neoplasia 2–3 after treatment with a large loop excision of the transformation zone: a retrospective study. 4) Factors affecting residual/recurrent cervical intraepithelial neoplasia after cervical conization with negative margins. 5) Predictors of Human papillomavirus (HPV) persistence after treatment of high grade cervical lesions; does cervical cytology have any prognostic value in primary HPV screening? 6) Human papillomavirus type-specific persistence and recurrence after treatment for cervical dysplasia. 7) Does the trend toward less deep excisions in LLETZ to minimize obstetric risk lead to less favorable oncological outcomes? 8) Persistent HPV-16 infection leads to recurrence of high-grade cervical intraepithelial neoplasia. 9) The Value of Partial HPV Genotyping After Conization of Cervical Dysplasias. 10) Clearance of human papillomavirus infection after successful conization in patients with cervical intraepithelial neoplasia. 11) Age-related changes in pre- and post-conization HPV genotype distribution among women with high-grade cervical intraepithelial neoplasia. 12) A randomized trial comparing limited-excision conisation to Large Loop Excision of the Transformation Zone (LLETZ) in cervical dysplasia patients. 13) Pre- and post-conization high-risk HPV testing predicts residual/recurrent disease in patients treated for CIN 2–3. 14) Risk factors of persistent HPV infection after treatment for high ‑ grade squamous intraepithelial lesion. 15) Human papillomavirus genotyping predicts residual/recurrent disease after local treatment for cervical intraepithelial neoplasia better than viral DNA testing. 16) Human Papillomavirus Test After Conization in Predicting Residual Disease in Subsequent Hysterectomy Specimens. 17) Residual lesions in uterine specimens after loop electrosurgical excision procedure in patients with CIN. 18) Recurrent high-grade cervical lesion after primary conization is associated with persistent human papillomavirus infection in Norway. 19) Persistence of human papillomavirus DNA in cervical lesions after treatment with diathermic large loop excision. 20) Human papillomavirus infection in patients with residual or recurrent cervical intraepithelial neoplasia. 21) Does LLETZ excision margin status predict residual disease in women who have undergone post-treatment cervical cytology and high-risk human papillomavirus testing? 22) Risk factors for human papillomavirus persistence among women undergoing cold-knife conization for treatment of high-grade cervical intraepithelial neoplasia. 23) Progression of cervical intraepithelial neoplasia grade 2 lesions among Japanese women harboring different genotype categories of high-risk human papillomaviruses. 24) The association of pre-conization high-risk HPV load and the persistence of HPV infection and persistence/recurrence of cervical intraepithelial neoplasia after conization. 25) Factors associated with HPV persistence after treatment for high-grade cervical intra-epithelial neoplasia with large loop excision of the transformation zone (LLETZ). 26) Human papillomavirus type-specific persistence and reappearance after successful conization in patients with cervical intraepithelial neoplasia. 27) Age, margin status, high-risk human papillomavirus and cytology independently predict recurrent high-grade cervical intraepithelial neoplasia up to 6 years after treatment. 28) Clinical Significance of the Interaction between Human Papillomavirus (HPV) Type 16 and Other High-Risk Human Papillomaviruses in Women with Cervical Intraepithelial Neoplasia (CIN) and Invasive Cervical Cancer.
