# Supplementary material for: Persistent HPV infection after conization of cervical intraepithelial neoplasia—— a systematic review and meta-analysis
Source: BMC Womens Health. 2023 May 3;23:216. doi: 10.1186/s12905-023-02360-w (PMC10155368; doi:10.1186/s12905-023-02360-w)

1. Role of human papillomavirus status after conization for high‐grade cervical intraepithelial neoplasia


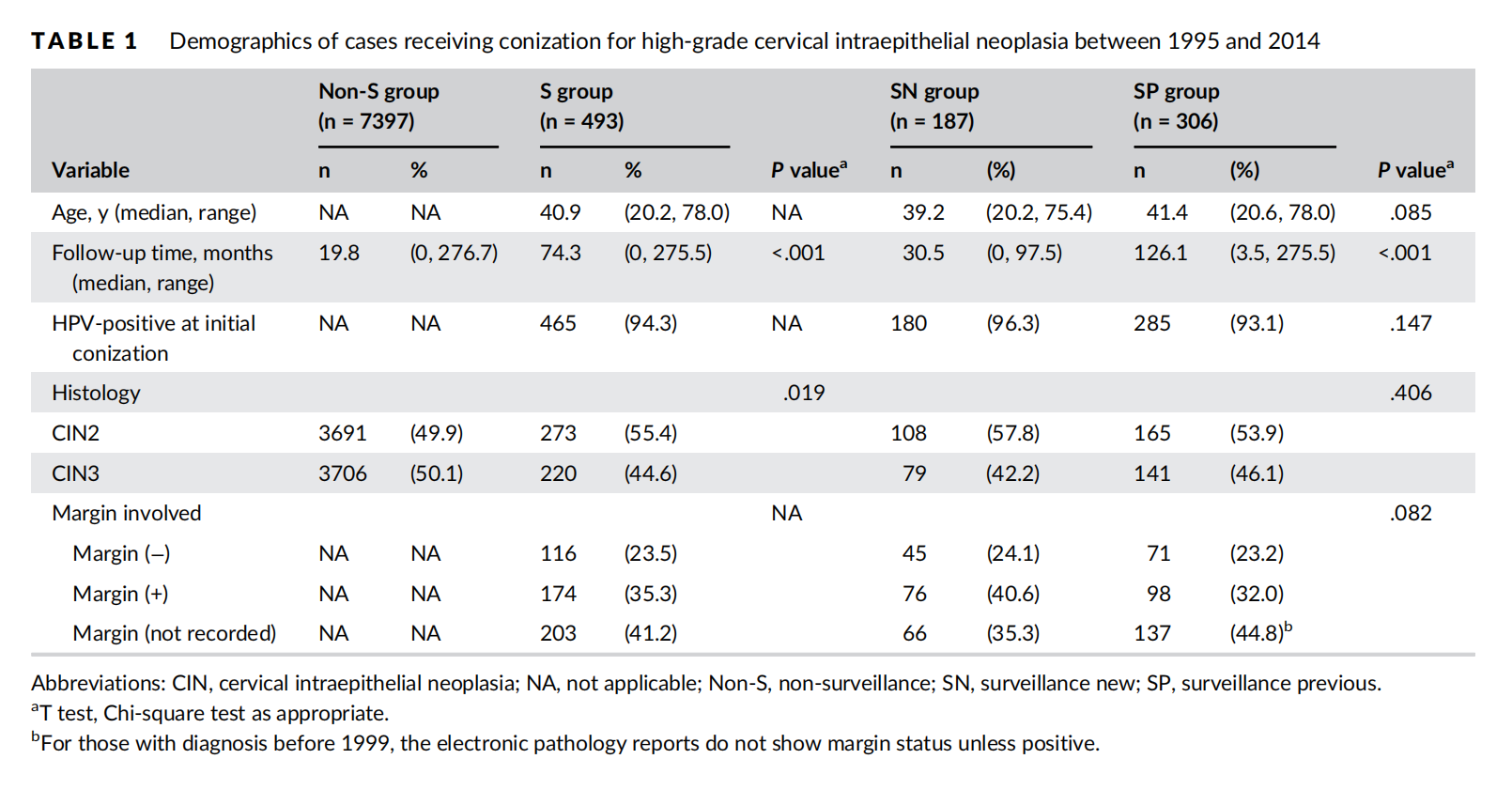


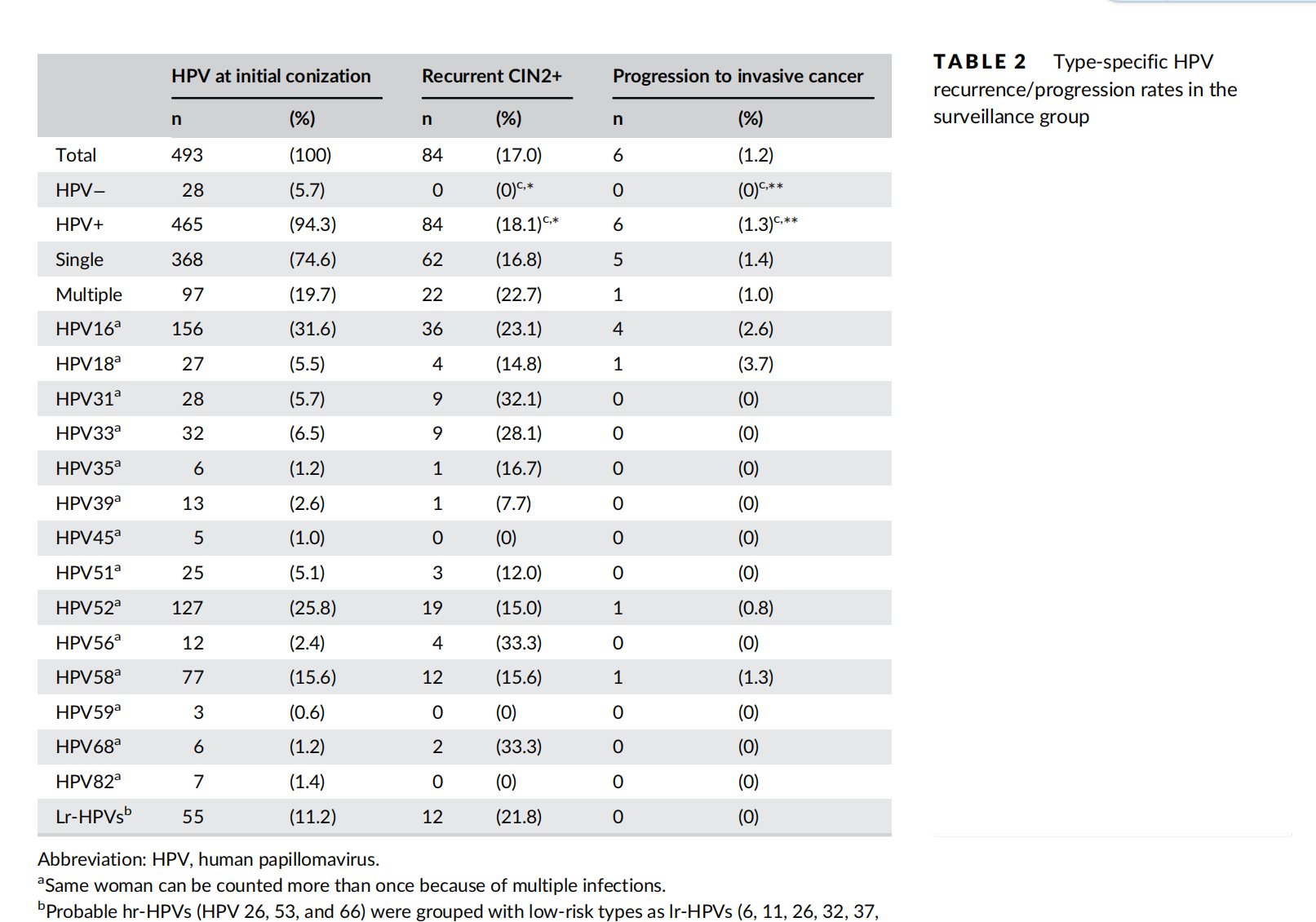


1. Risk factors for type-specific persistence of high-risk human papillomavirus and residual/recurrent cervical intraepithelial neoplasia after surgical treatment


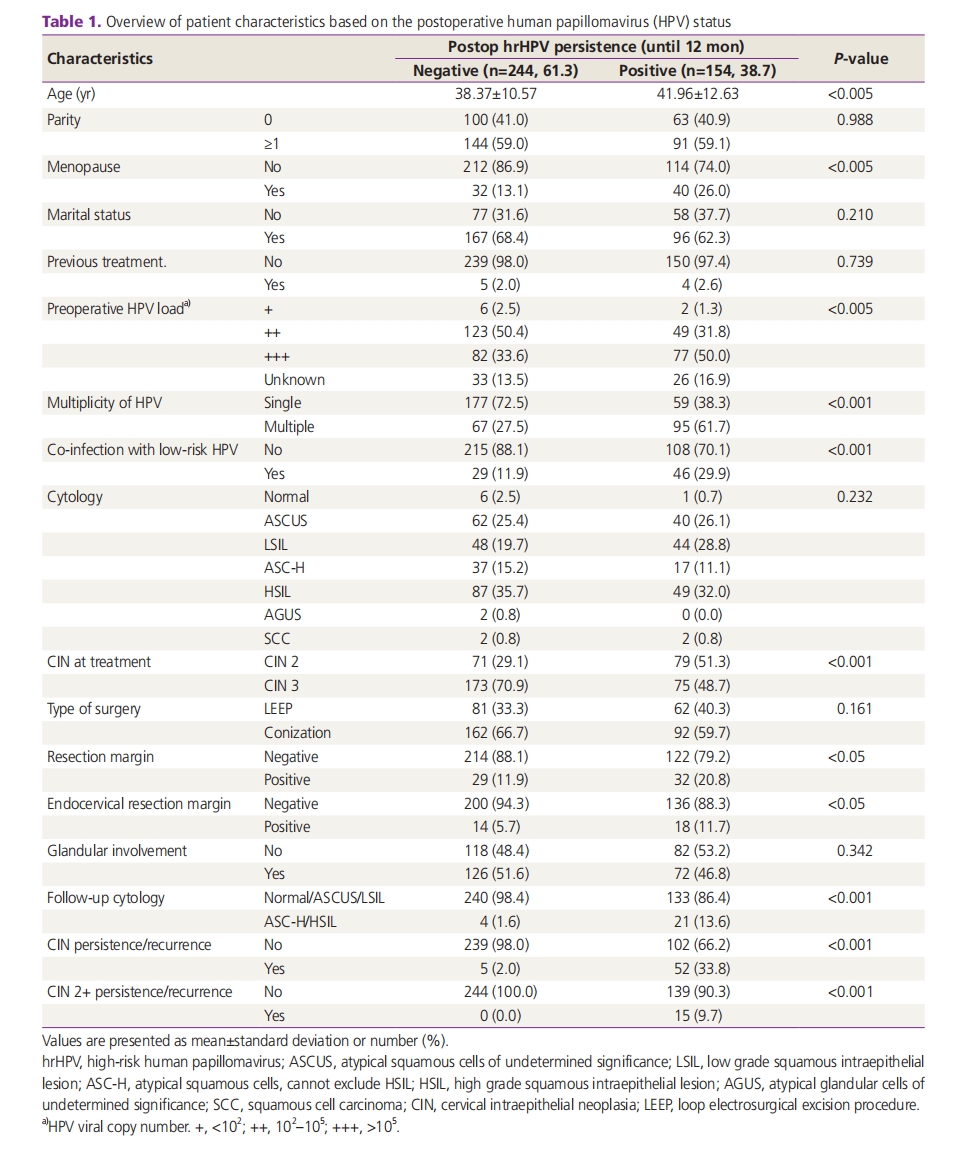


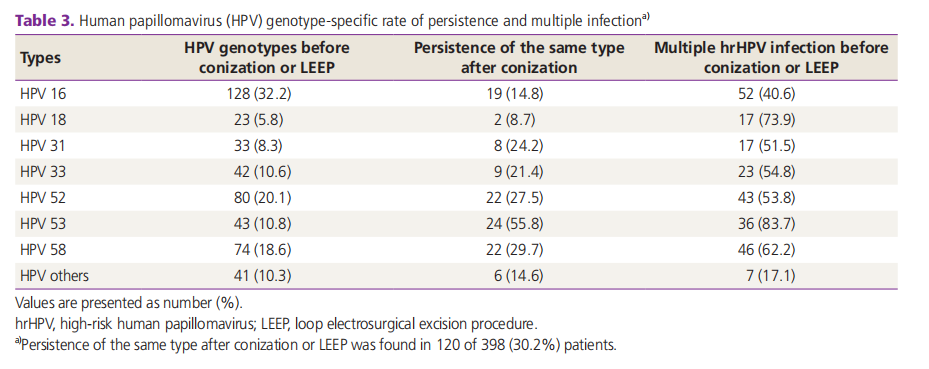


1. Long‐term predictors of residual or recurrent cervical intraepithelial neoplasia 2–3 after treatment with a large loop excision of the transformation zone: a retrospective study


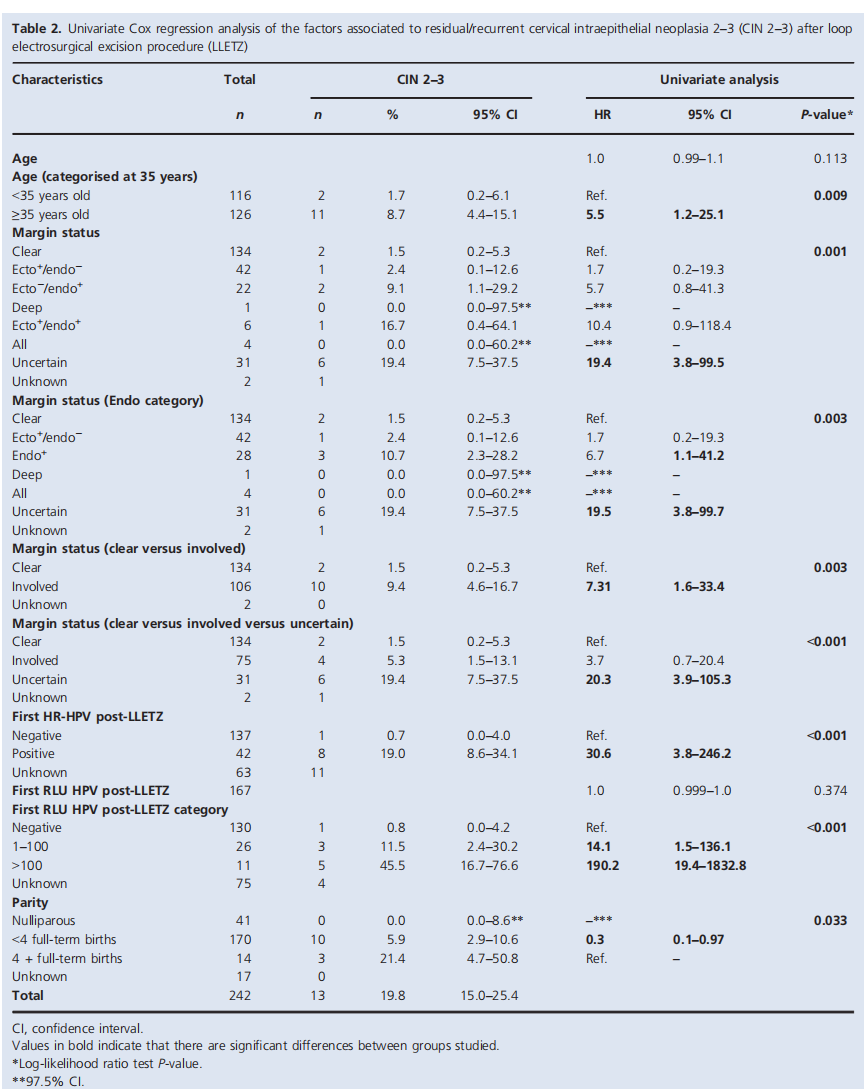


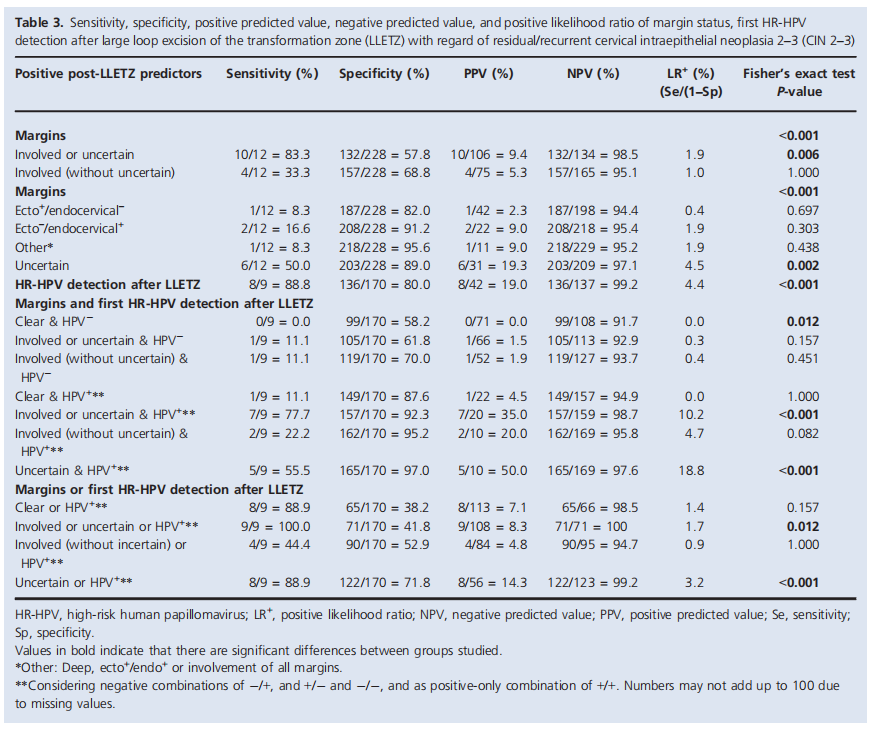


1. Factors affecting residual/recurrent cervical intraepithelial neoplasia after cervical conization with negative margins


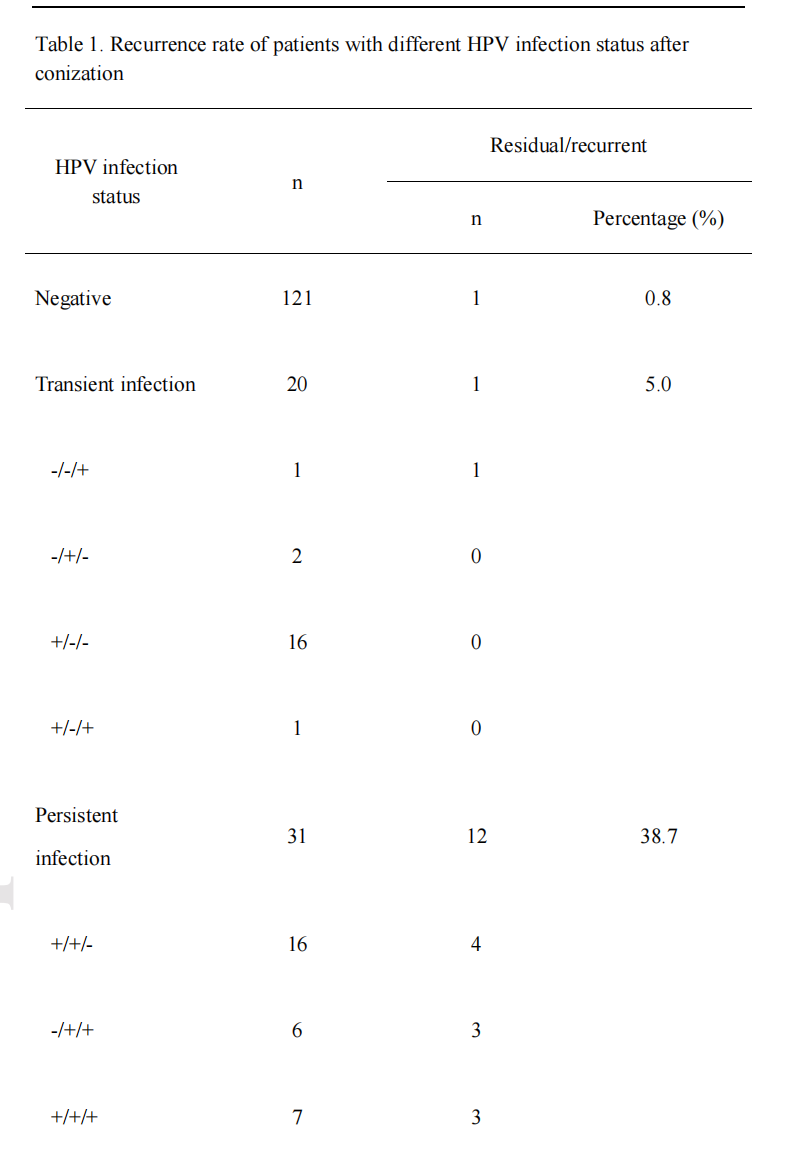


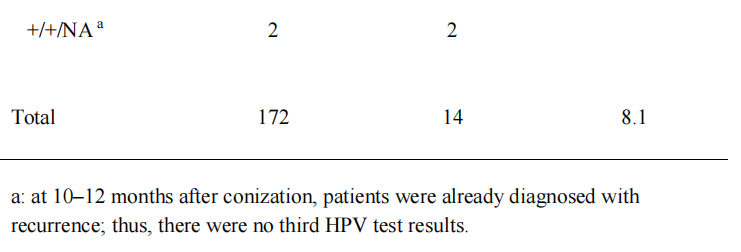


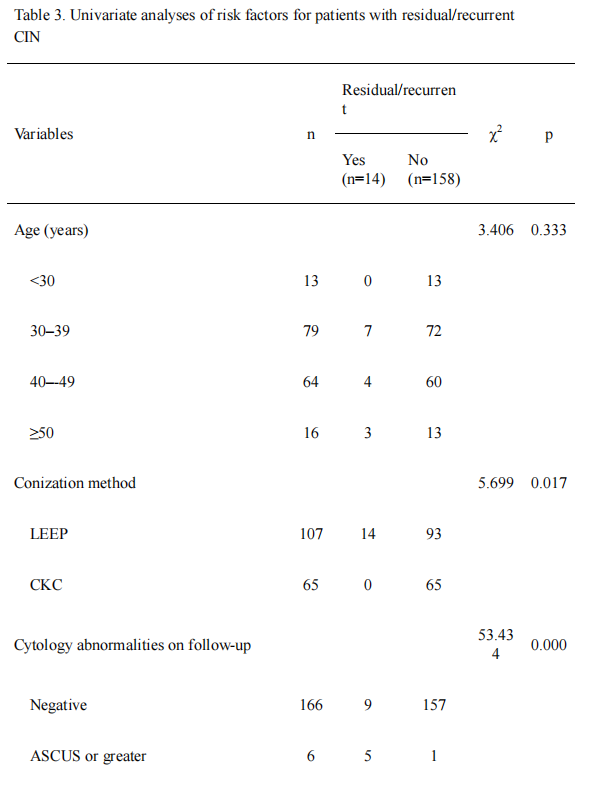


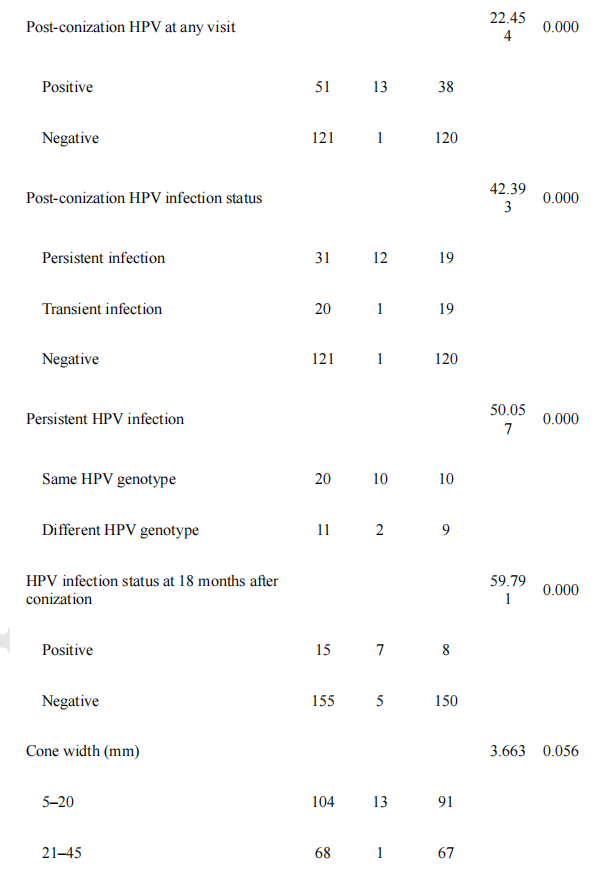


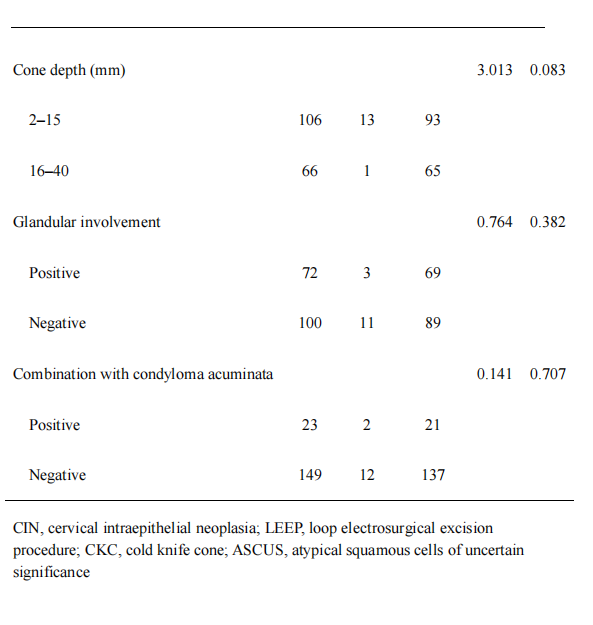


1. Predictors of Human papillomavirus (HPV) persistence after treatment of high grade cervical lesions; does cervical cytology have any prognostic value in primary HPV screening?


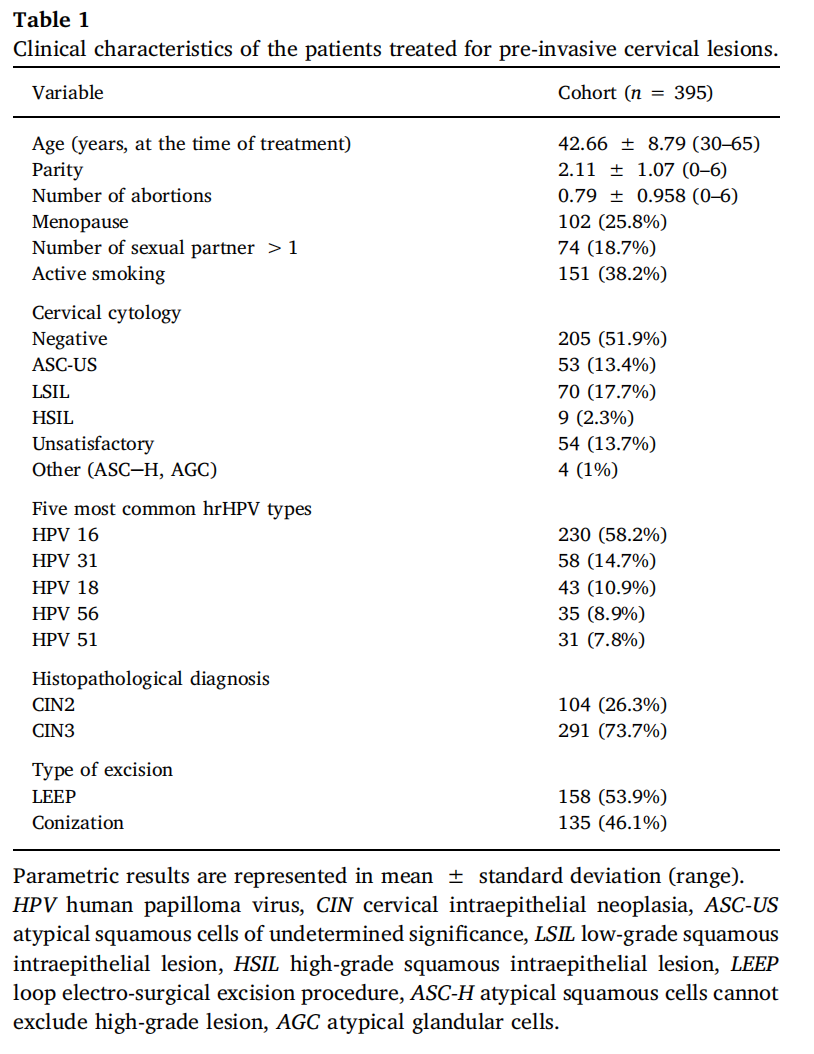


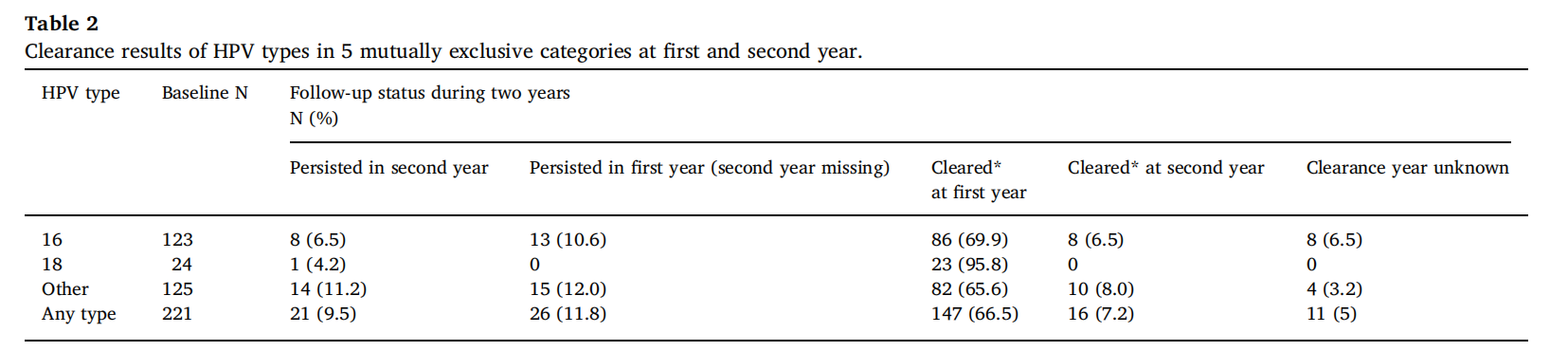


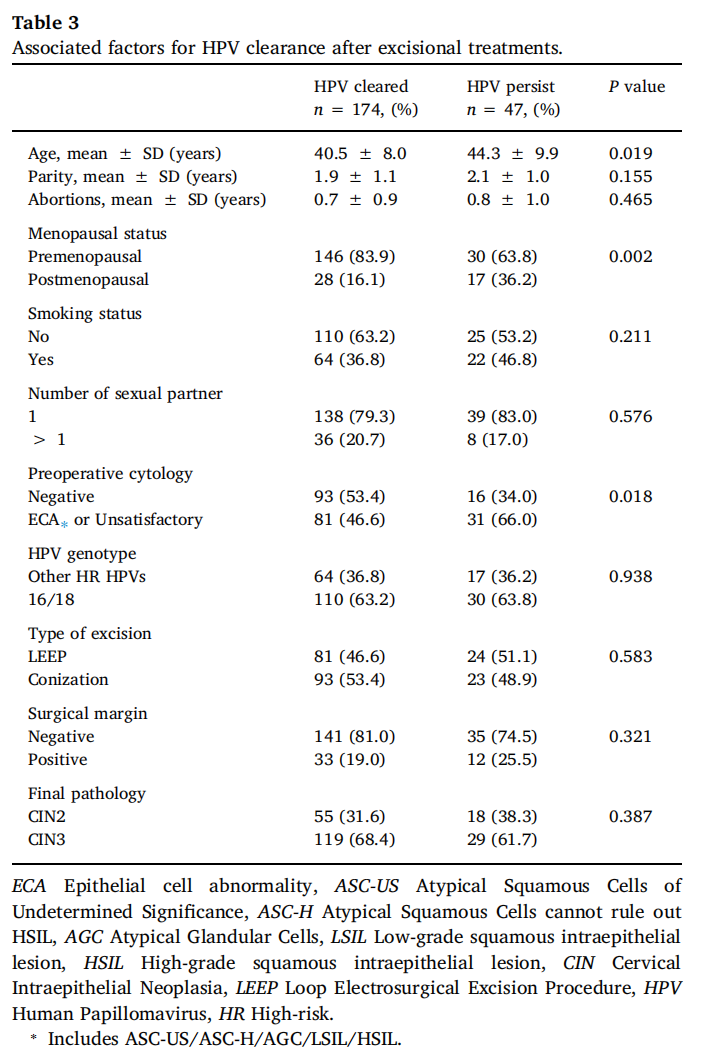


1. Human papillomavirus type-specific persistence and recurrence after treatment for cervical dysplasia


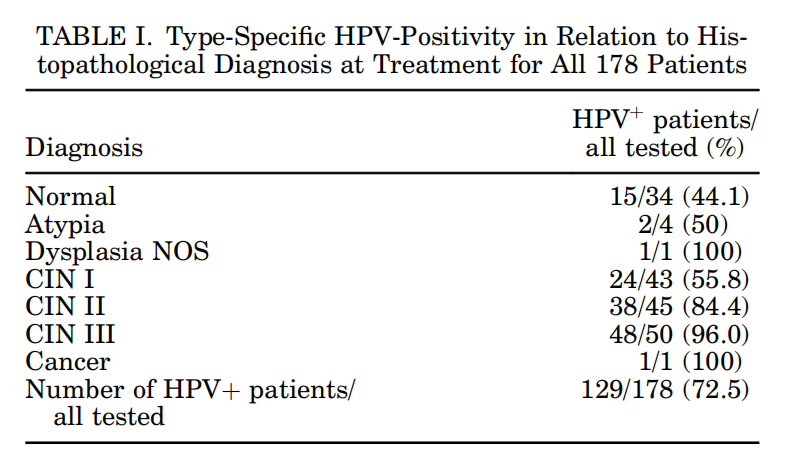


1. Does the trend toward less deep excisions in LLETZ to minimize obstetric risk lead to less favorable oncological outcomes?


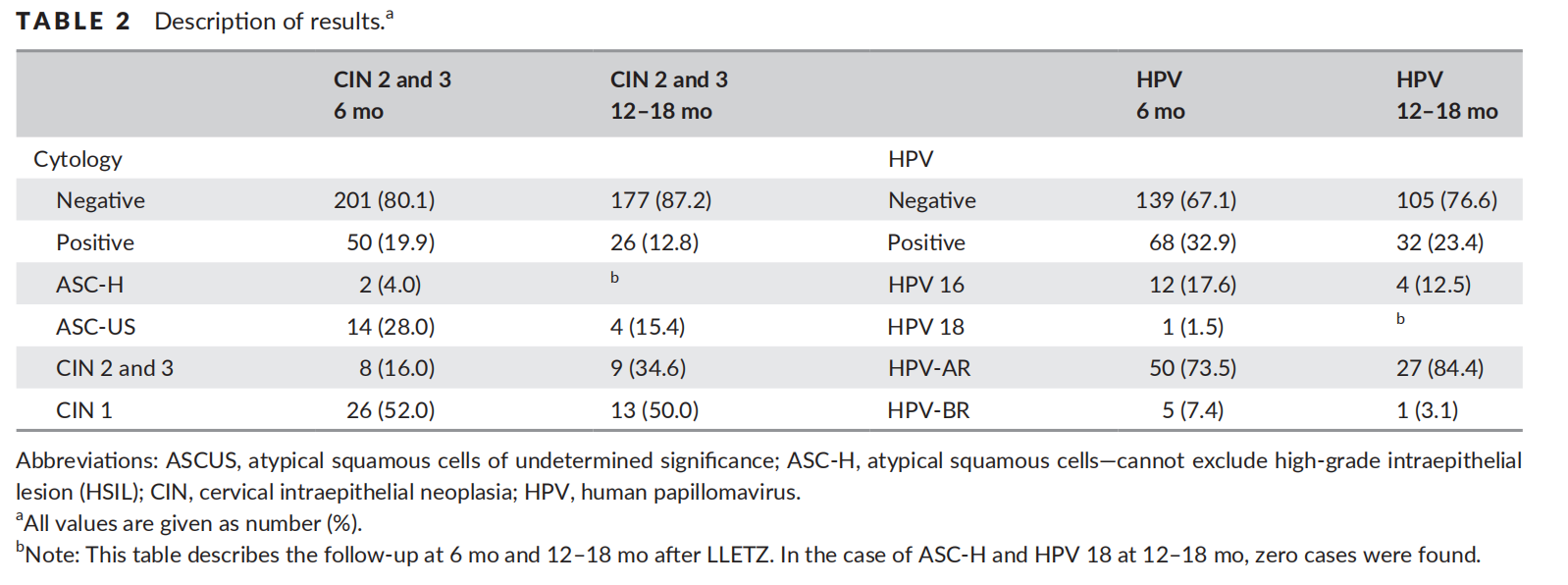


1. Persistent HPV-16 infection leads to recurrence of high-grade cervical intraepithelial neoplasia


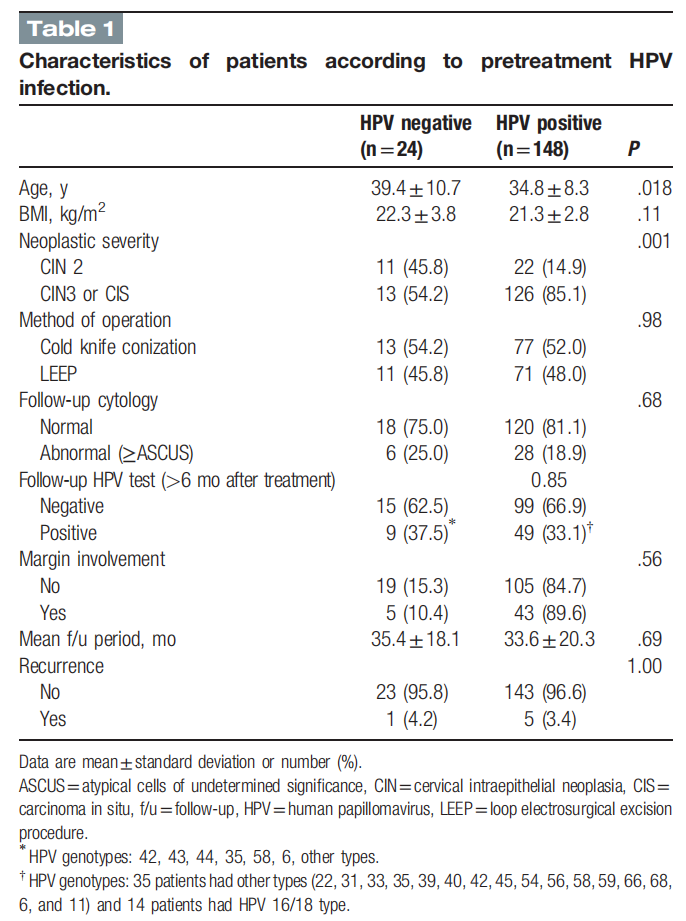


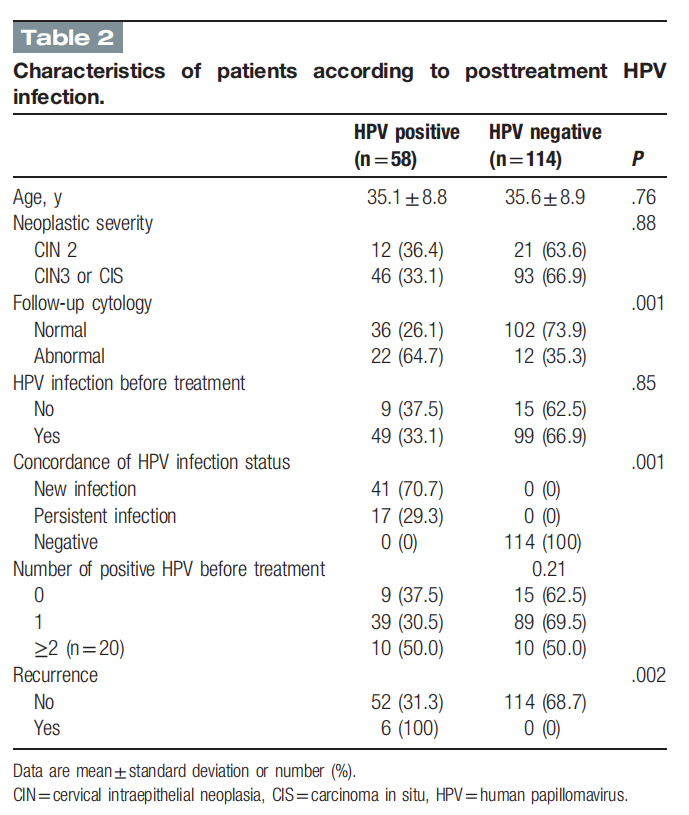


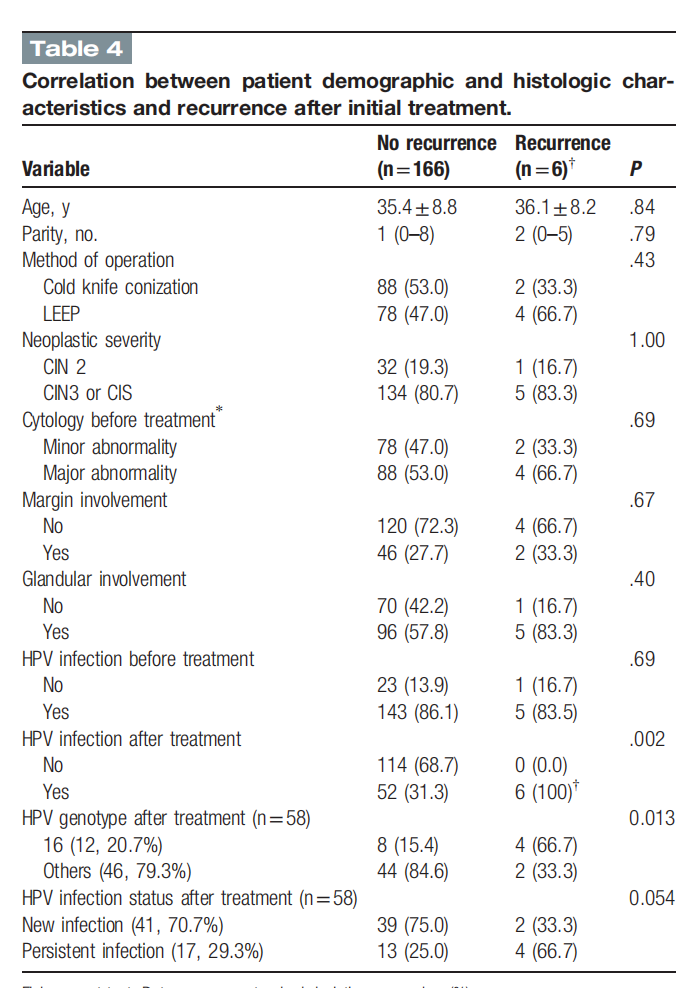


1. The Value of Partial HPV Genotyping After Conization of Cervical Dysplasias


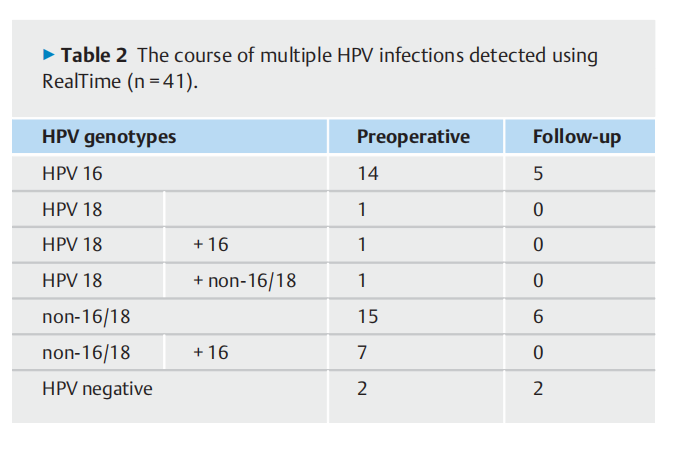


1. Clearance of human papillomavirus infection after successful conization in patients with cervical intraepithelial neoplasia


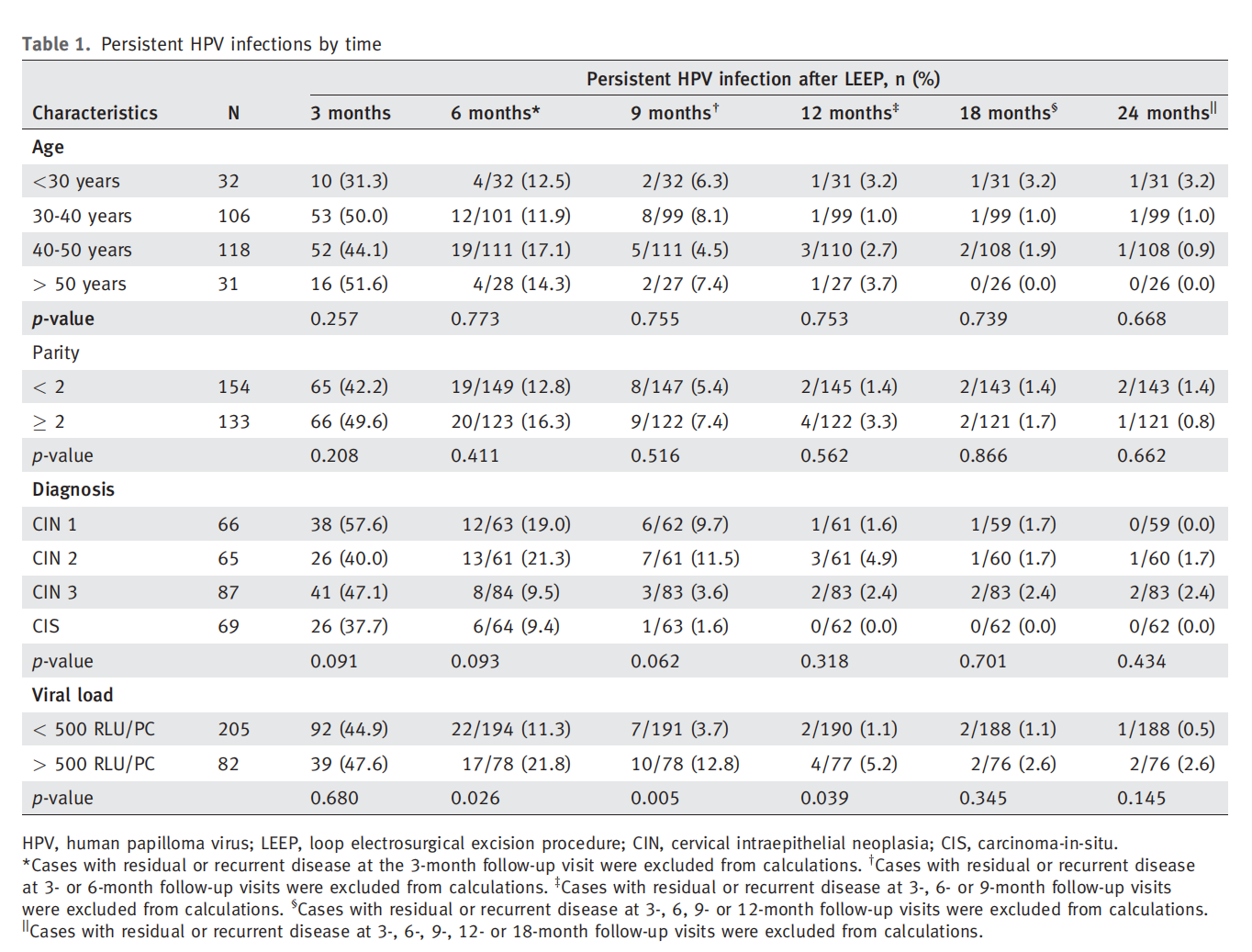


1. Age-related changes in pre- and post-conization HPV genotype distribution among women with high-grade cervical intraepithelial neoplasia


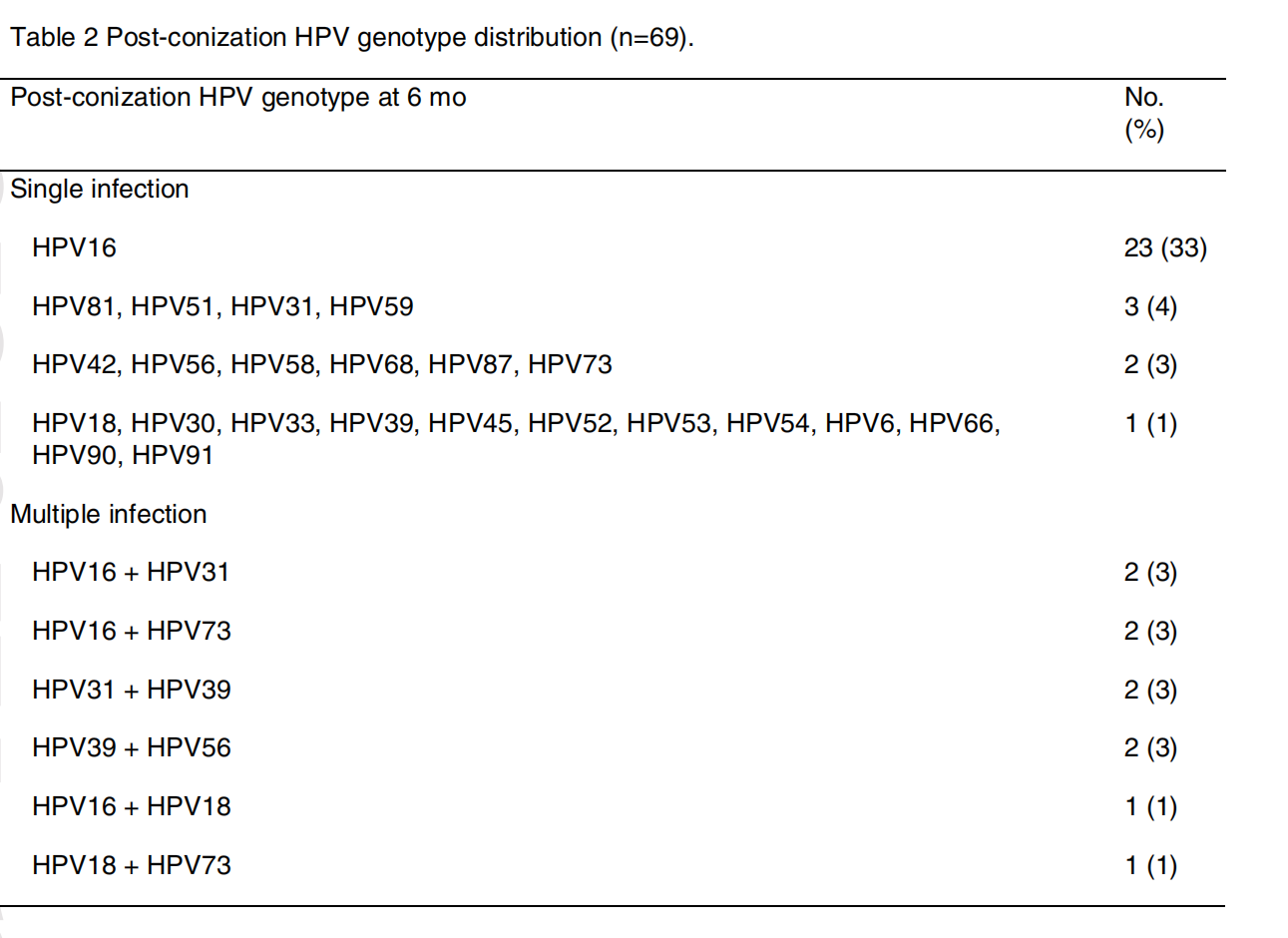


1. A randomized trial comparing limited-excision conisation to Large Loop Excision of the Transformation Zone (LLETZ) in cervical dysplasia patients


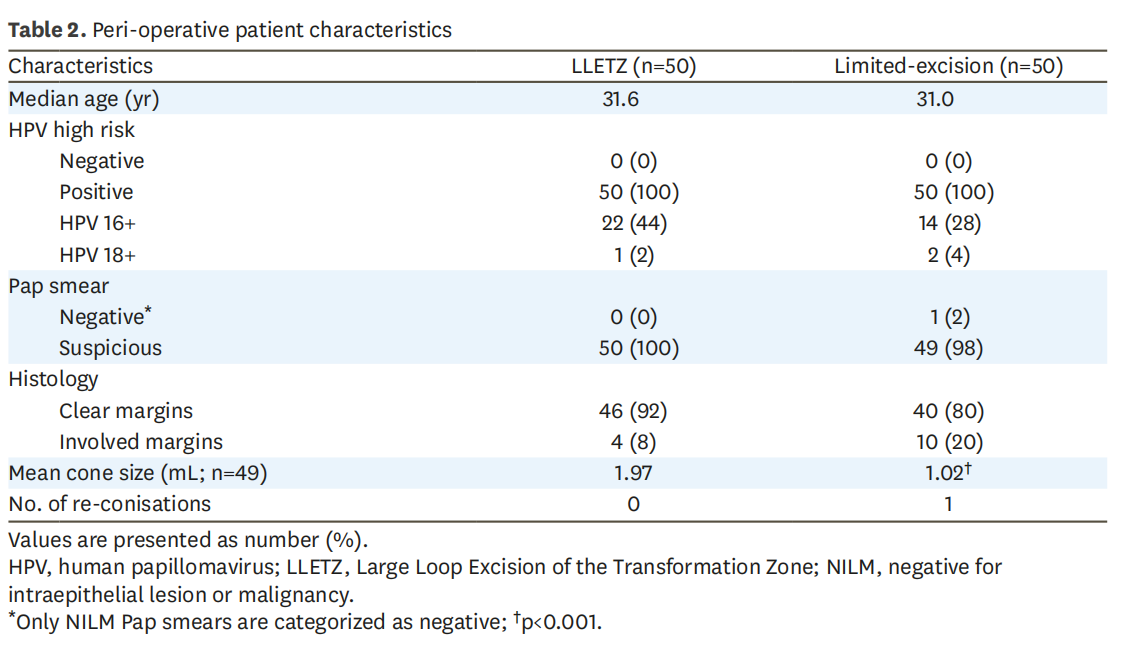


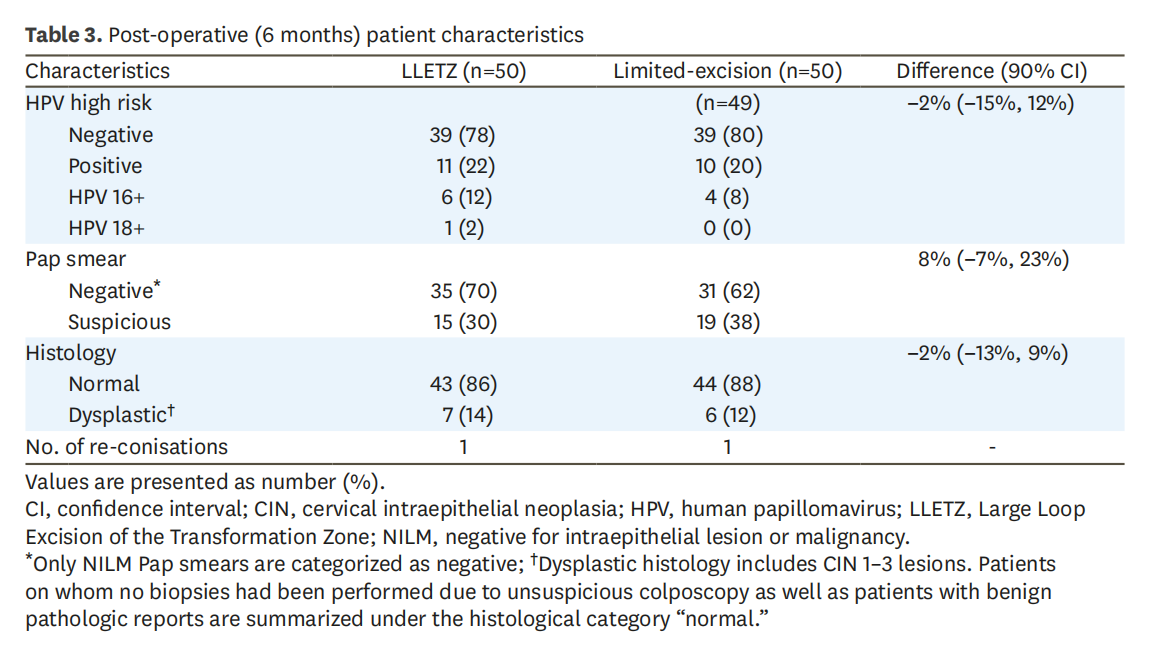


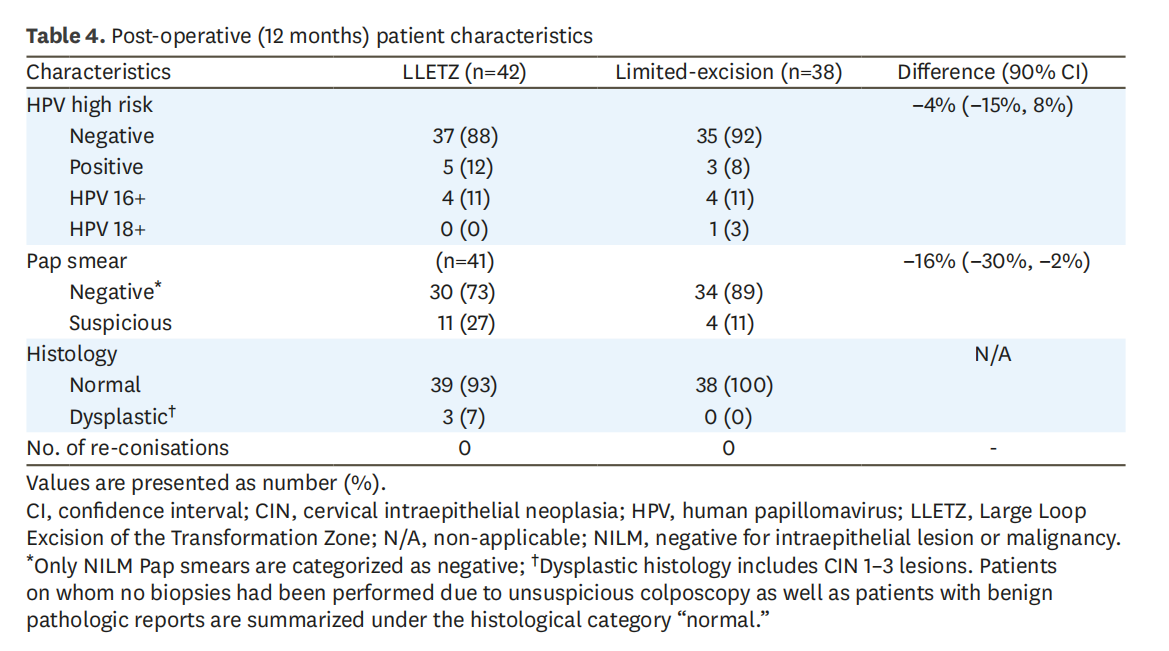


1. Pre- and post-conization high-risk HPV testing predicts residual/recurrent disease in patients treated for CIN 2–3


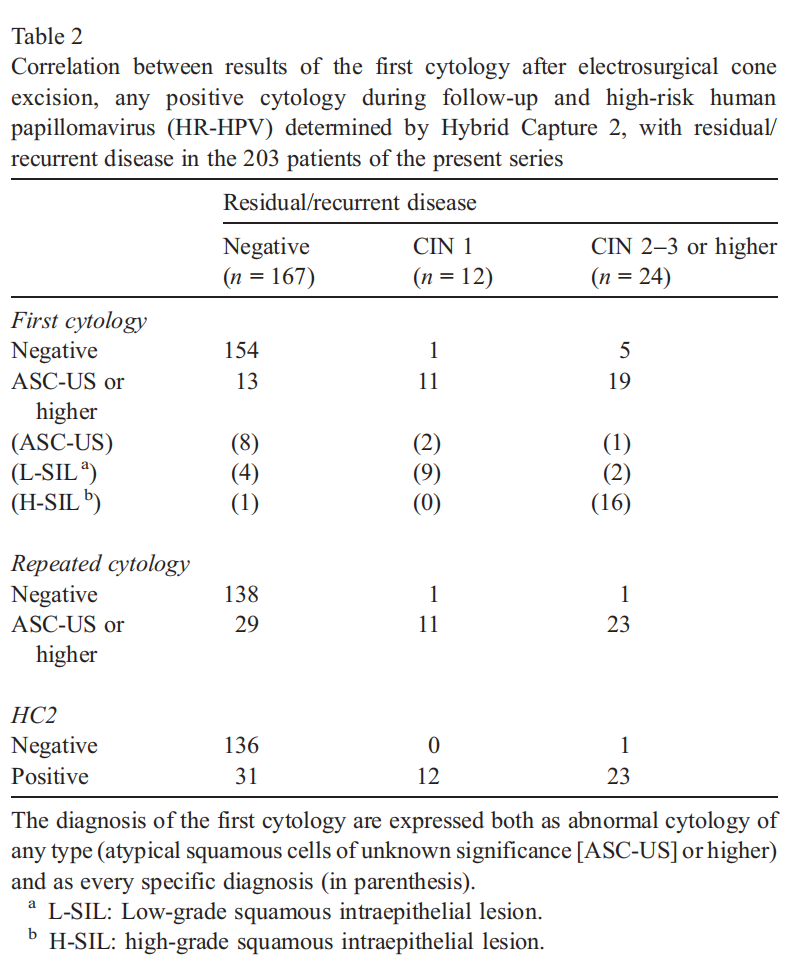


1. Risk factors of persistent HPV infection after treatment for high ‑ grade squamous intraepithelial lesion


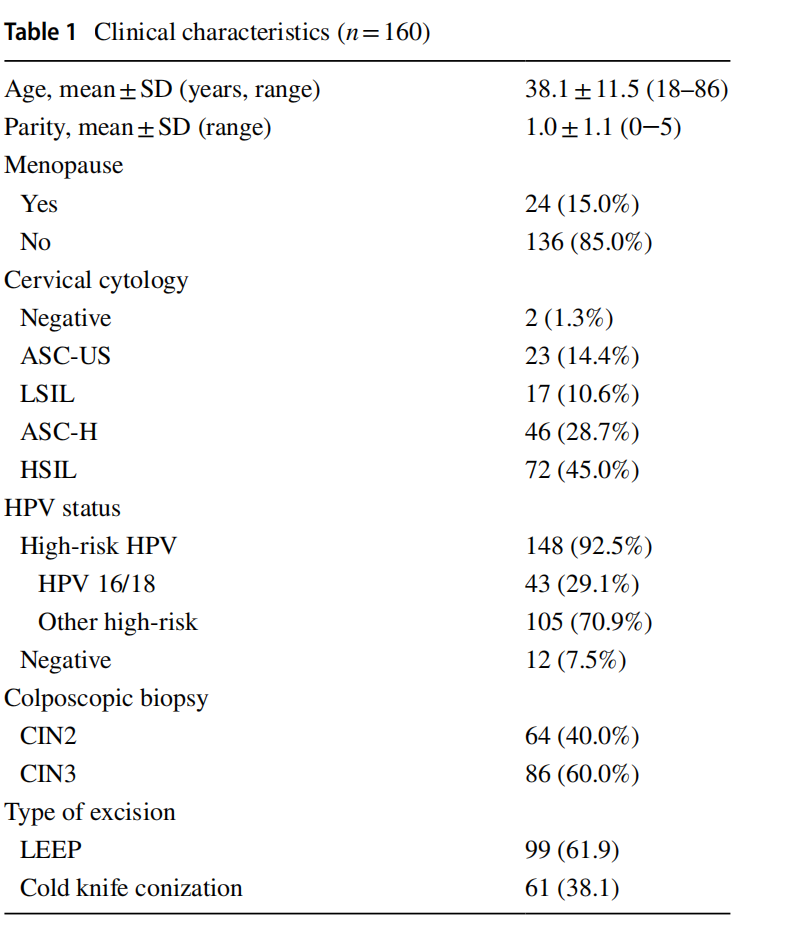


1. Human papillomavirus genotyping predicts residual/recurrent disease after local treatment for cervical intraepithelial neoplasia better than viral DNA testing


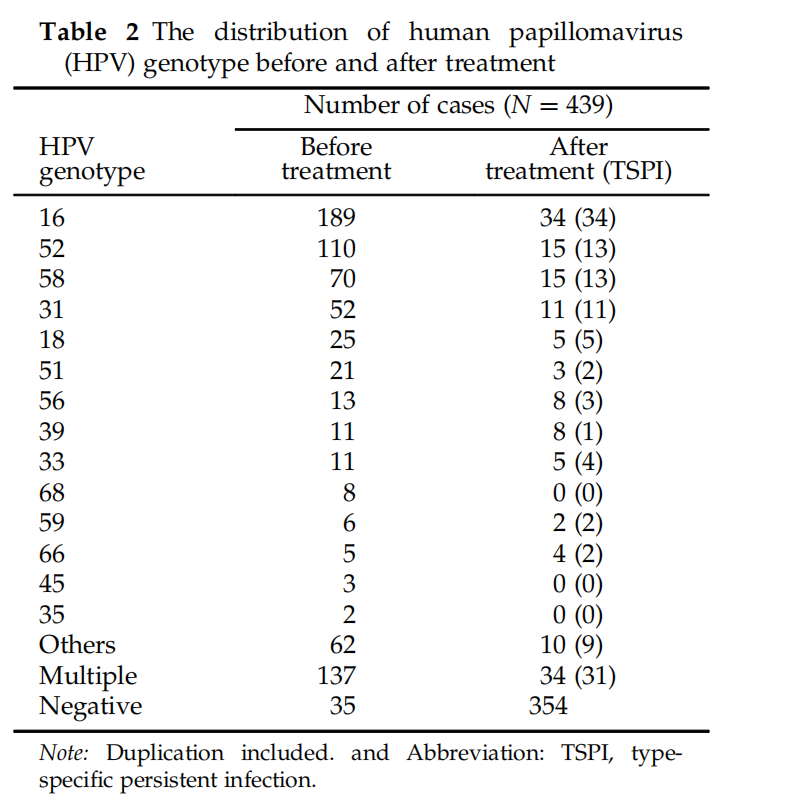


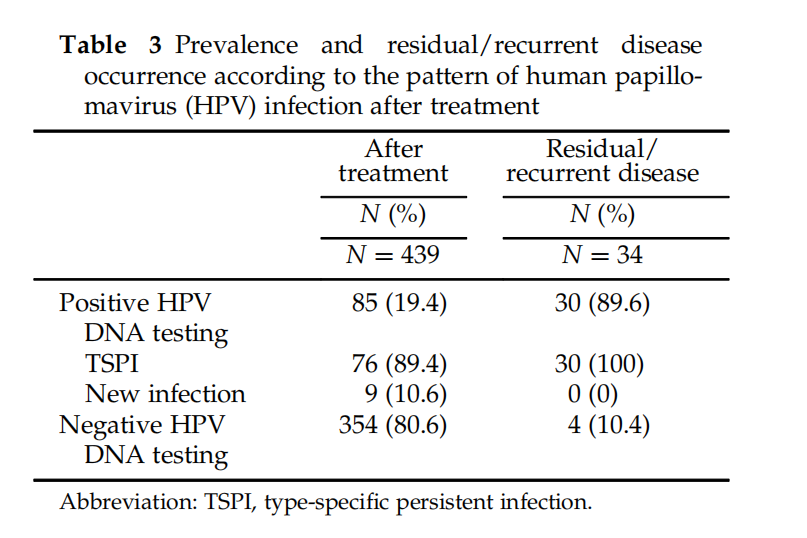


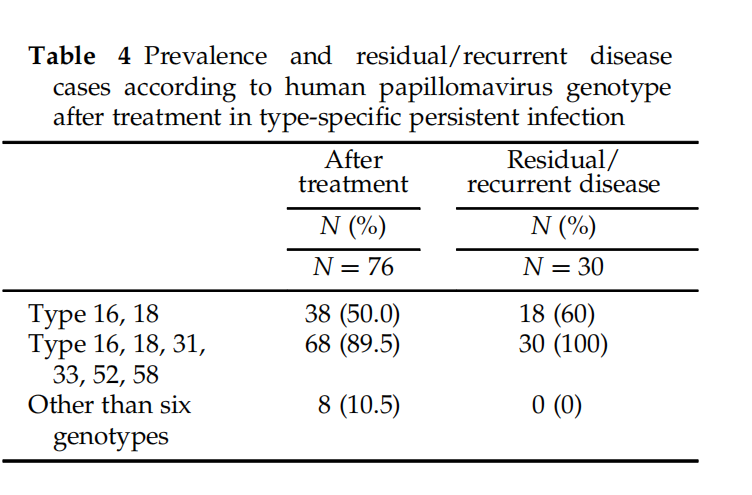


1. Human Papillomavirus Test After Conization in Predicting Residual Disease in Subsequent Hysterectomy Specimens


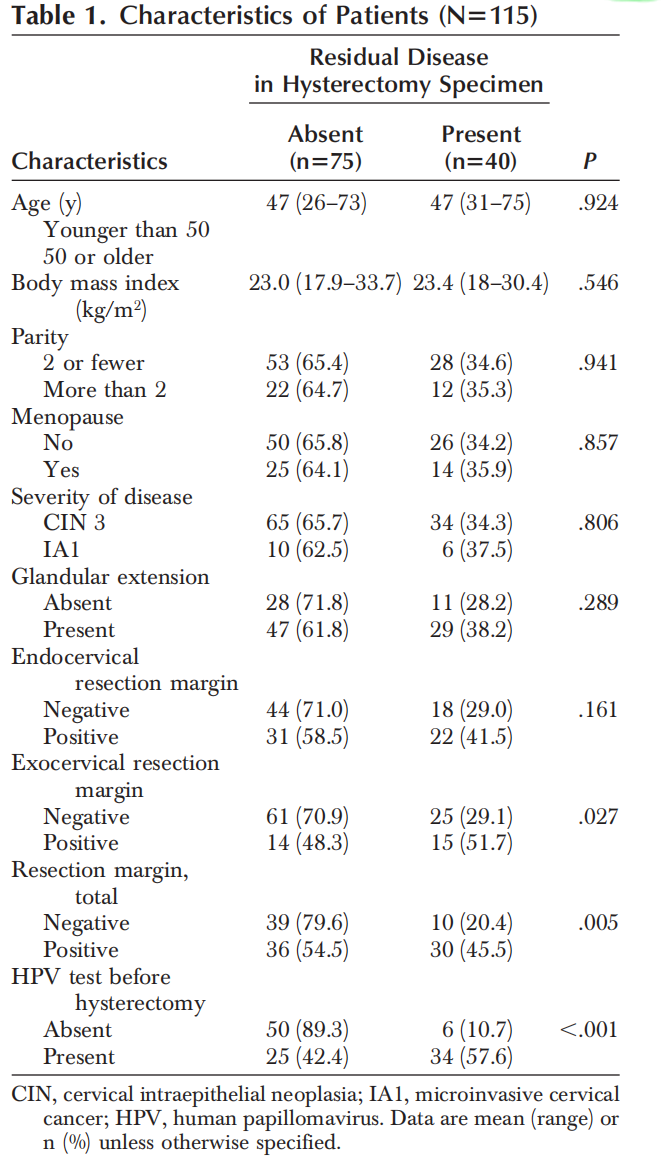


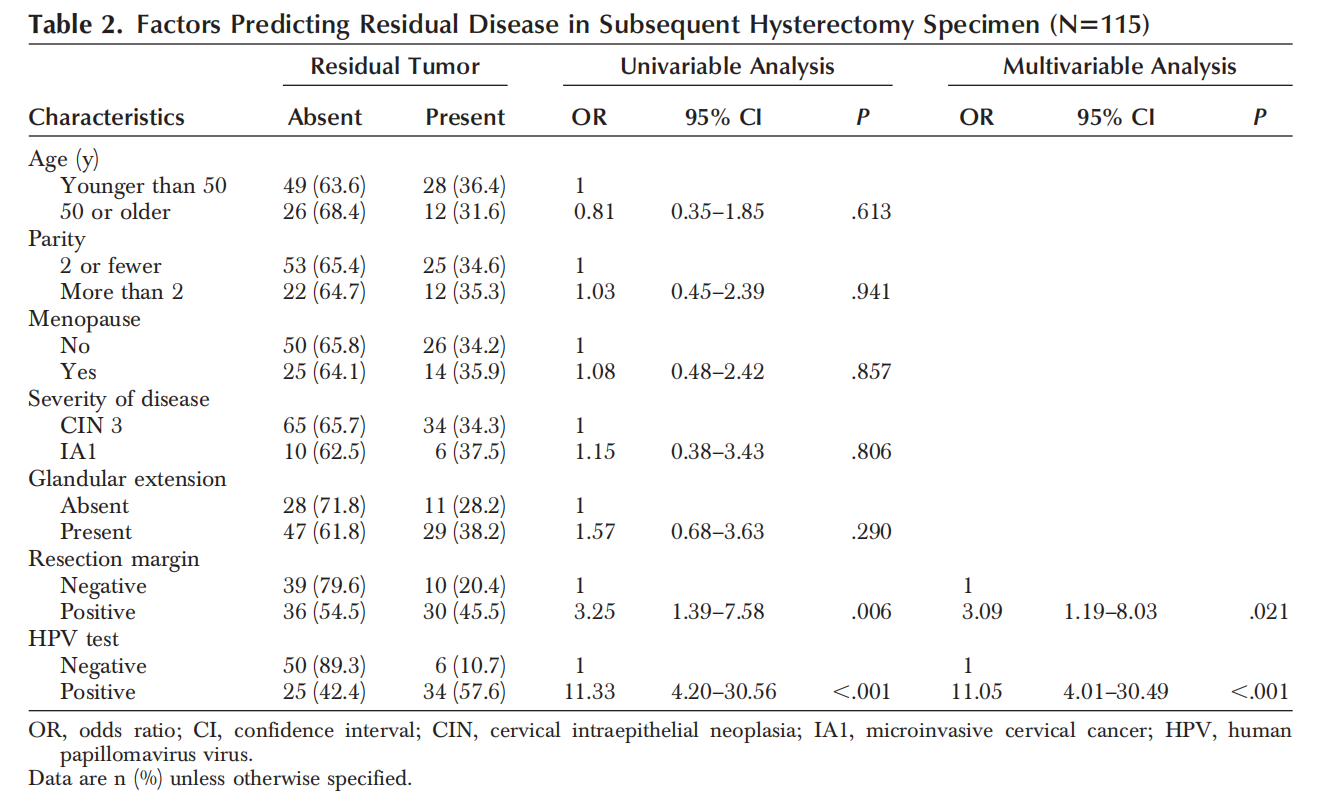


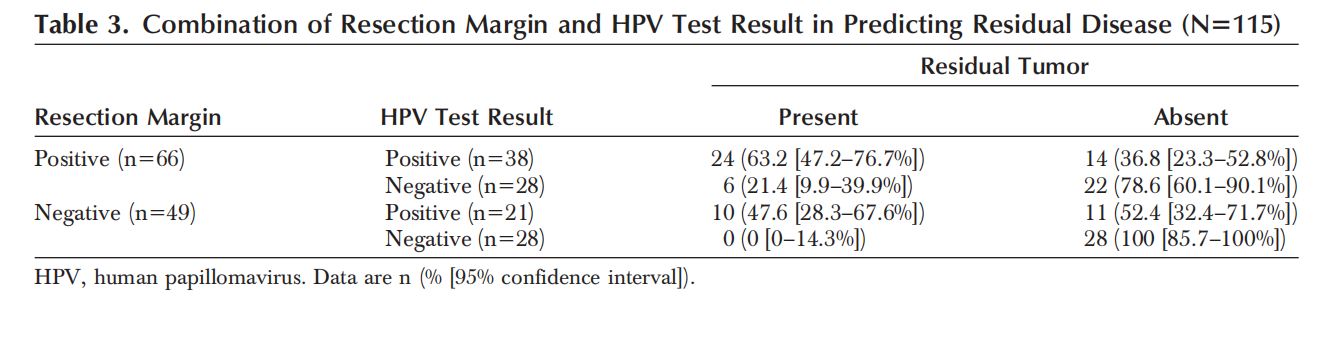


1. Residual lesions in uterine specimens after loop electrosurgical excision procedure in patients with CIN


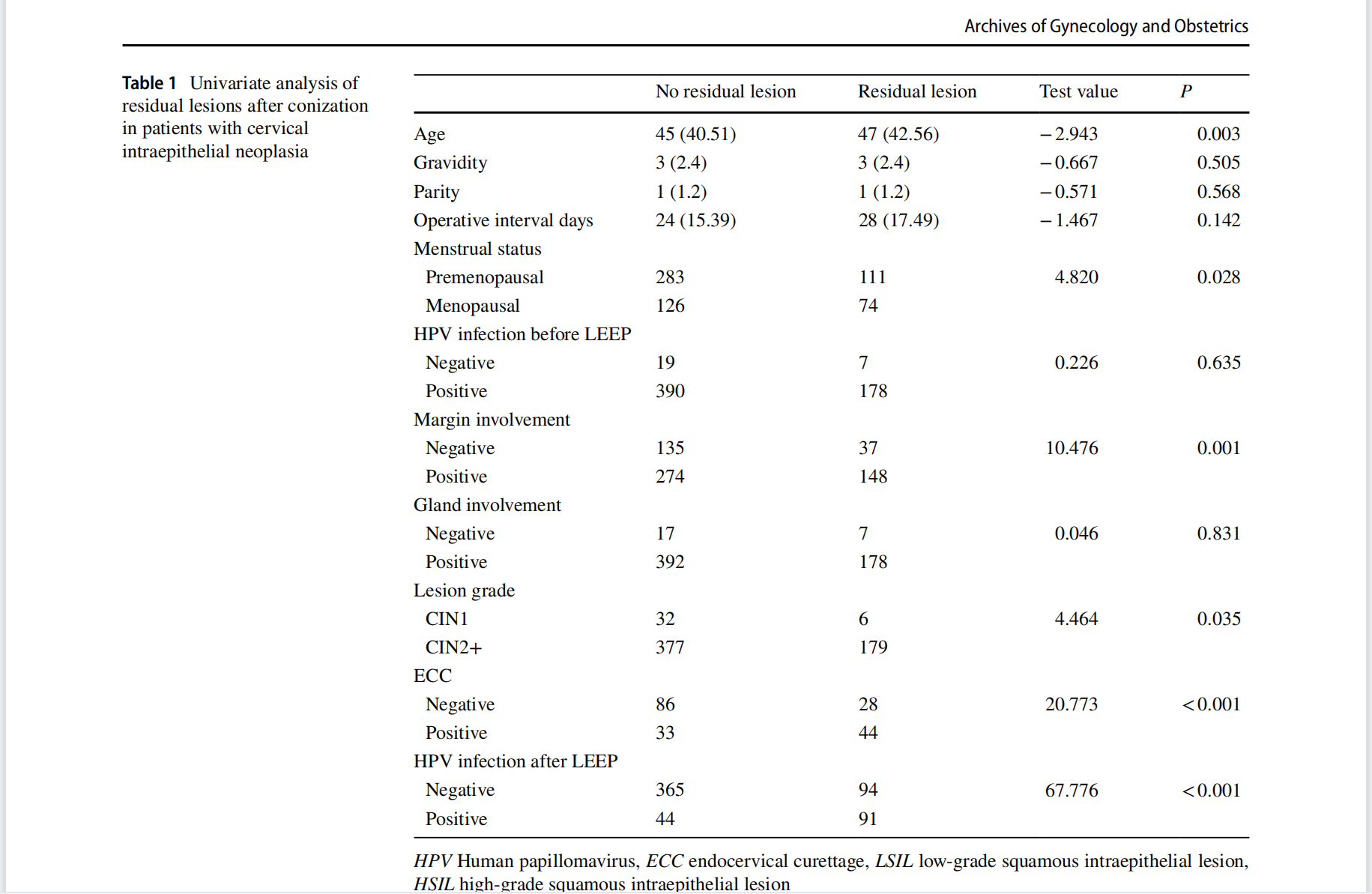


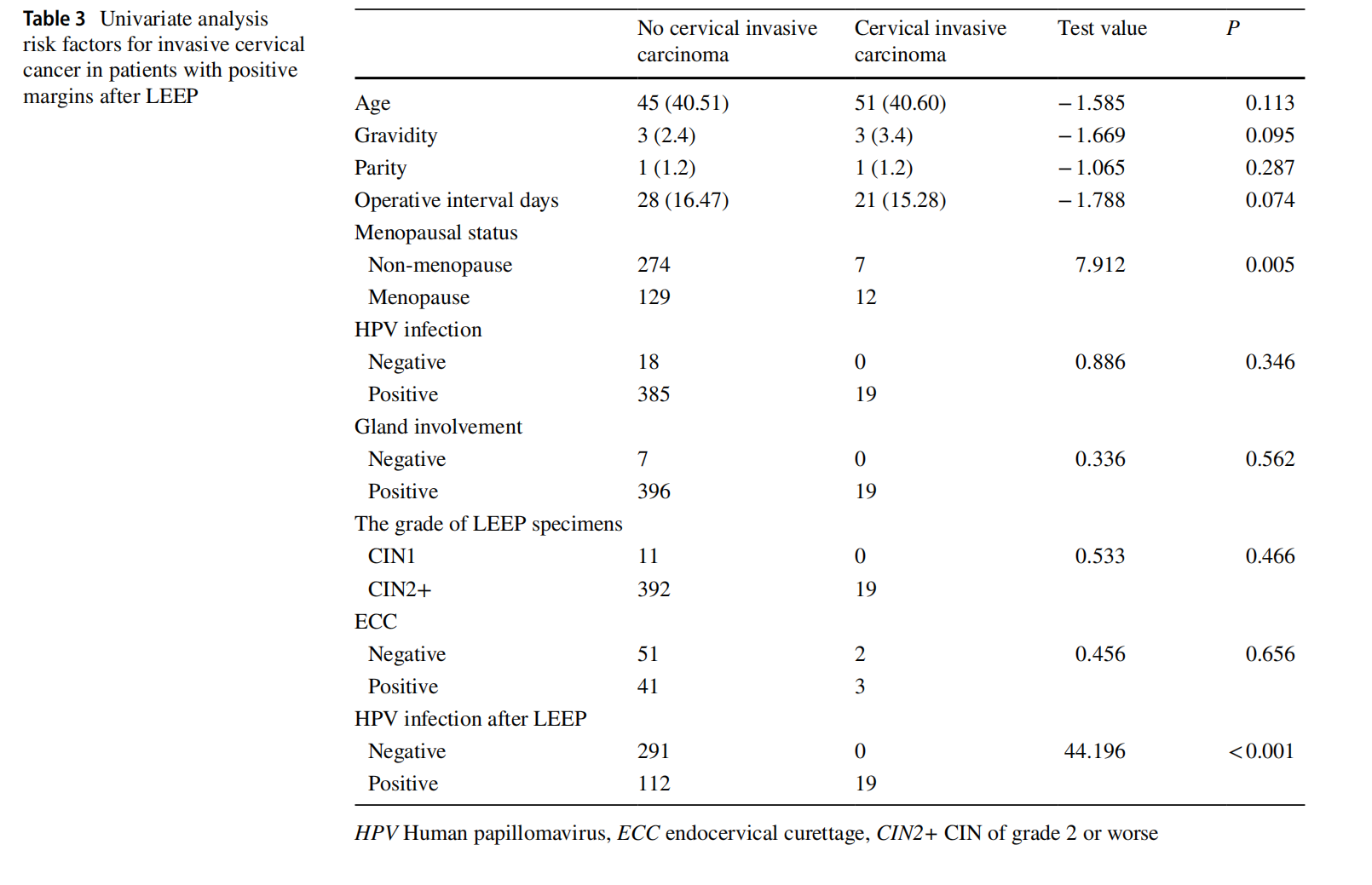


1. Recurrent high-grade cervical lesion after primary conization is associated with persistent human papillomavirus infection in Norway


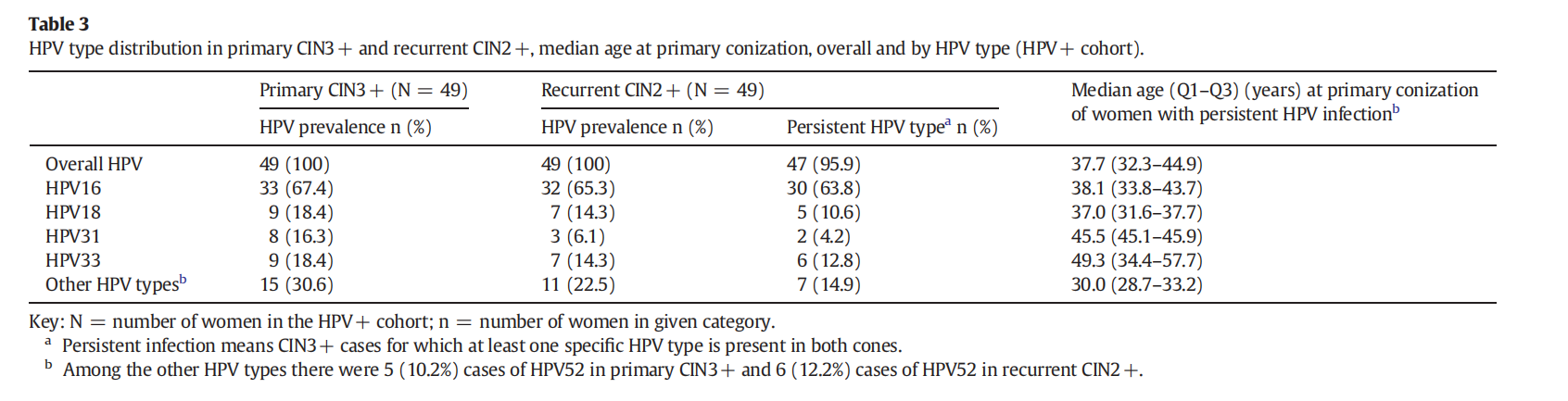


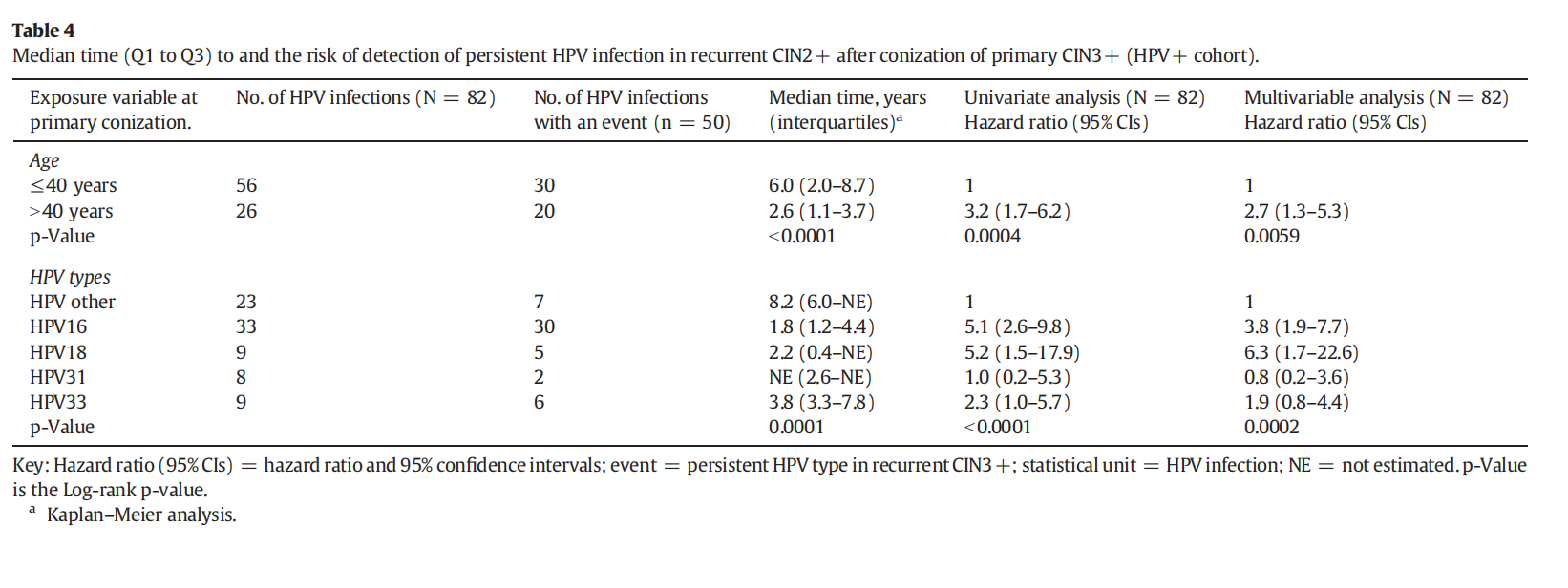


1. Persistence of human papillomavirus DNA in cervical lesions after treatment with diathermic large loop excision


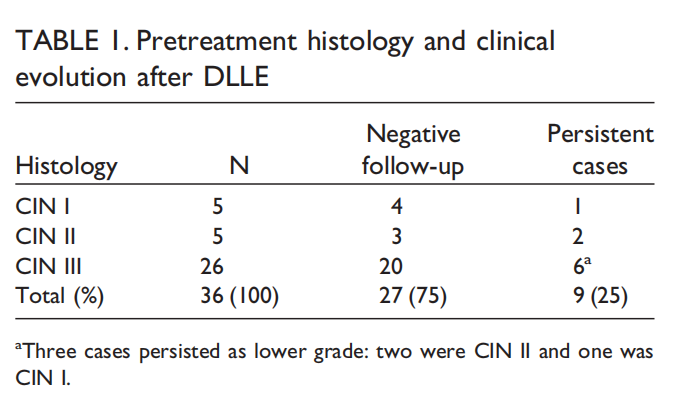


1. Human papillomavirus infection in patients with residual or recurrent cervical intraepithelial neoplasia


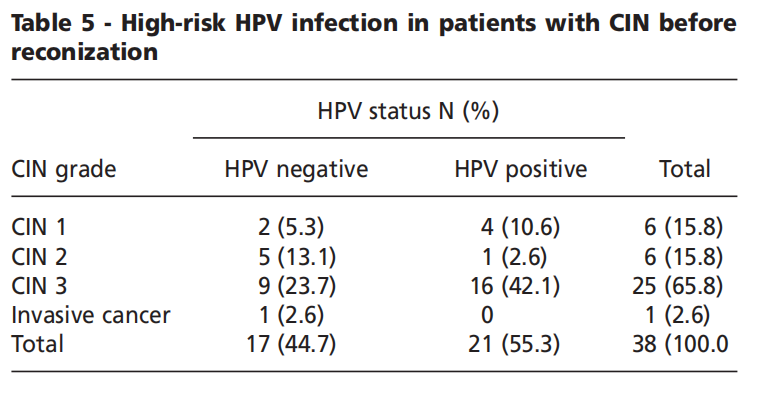


1. Does LLETZ excision margin status predict residual disease in women who have undergone post-treatment cervical cytology and high-risk human papillomavirus testing?


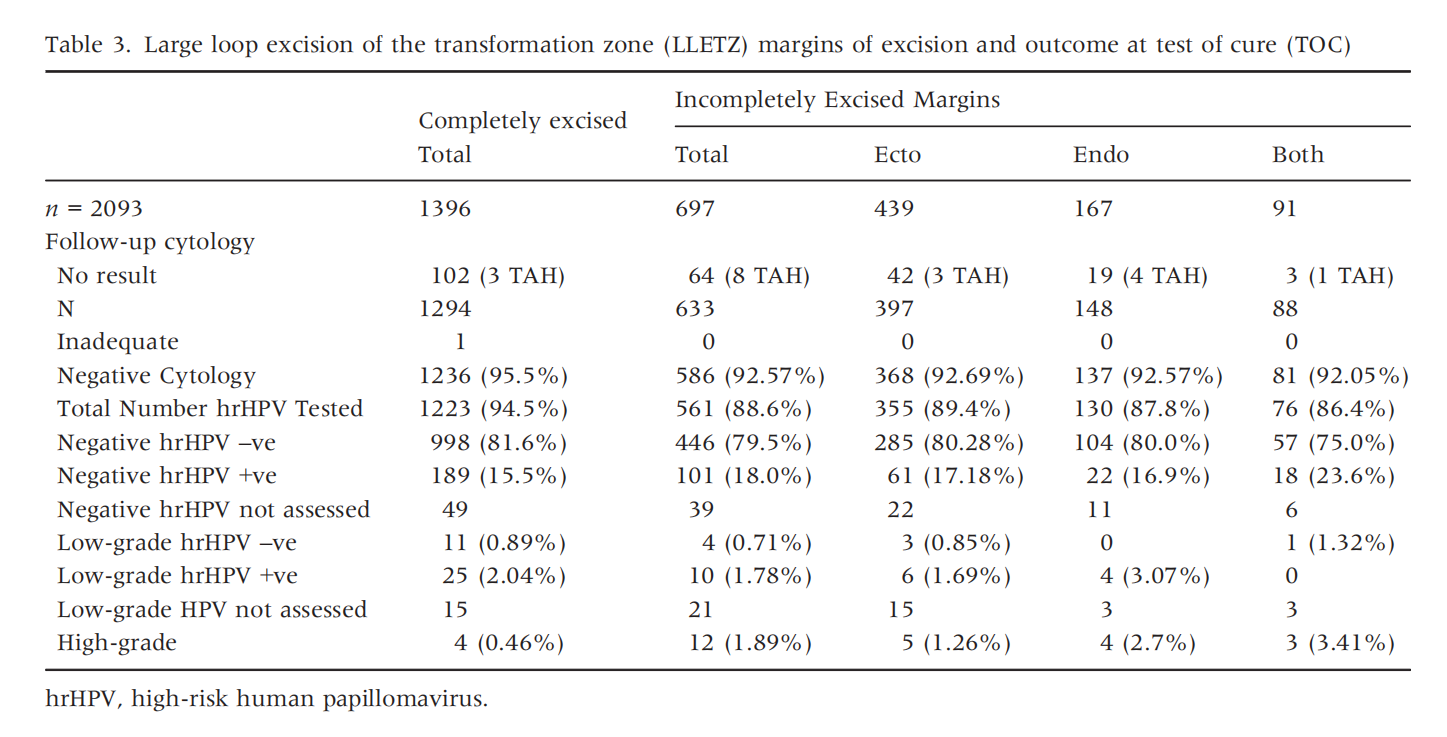


1. Risk factors for human papillomavirus persistence among women undergoing cold-knife conization for treatment of high-grade cervical intraepithelial neoplasia


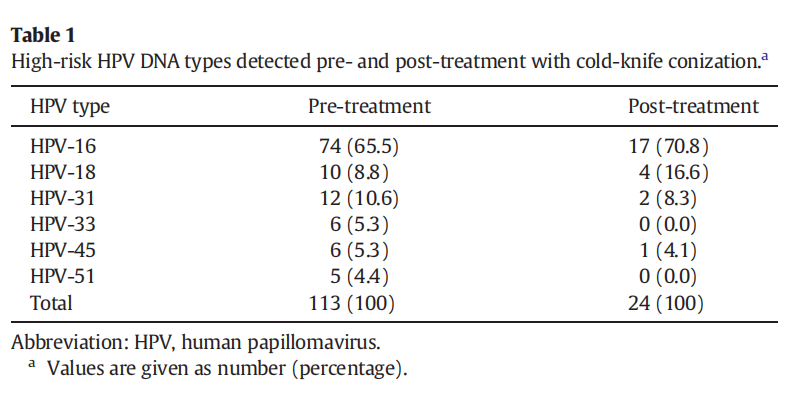


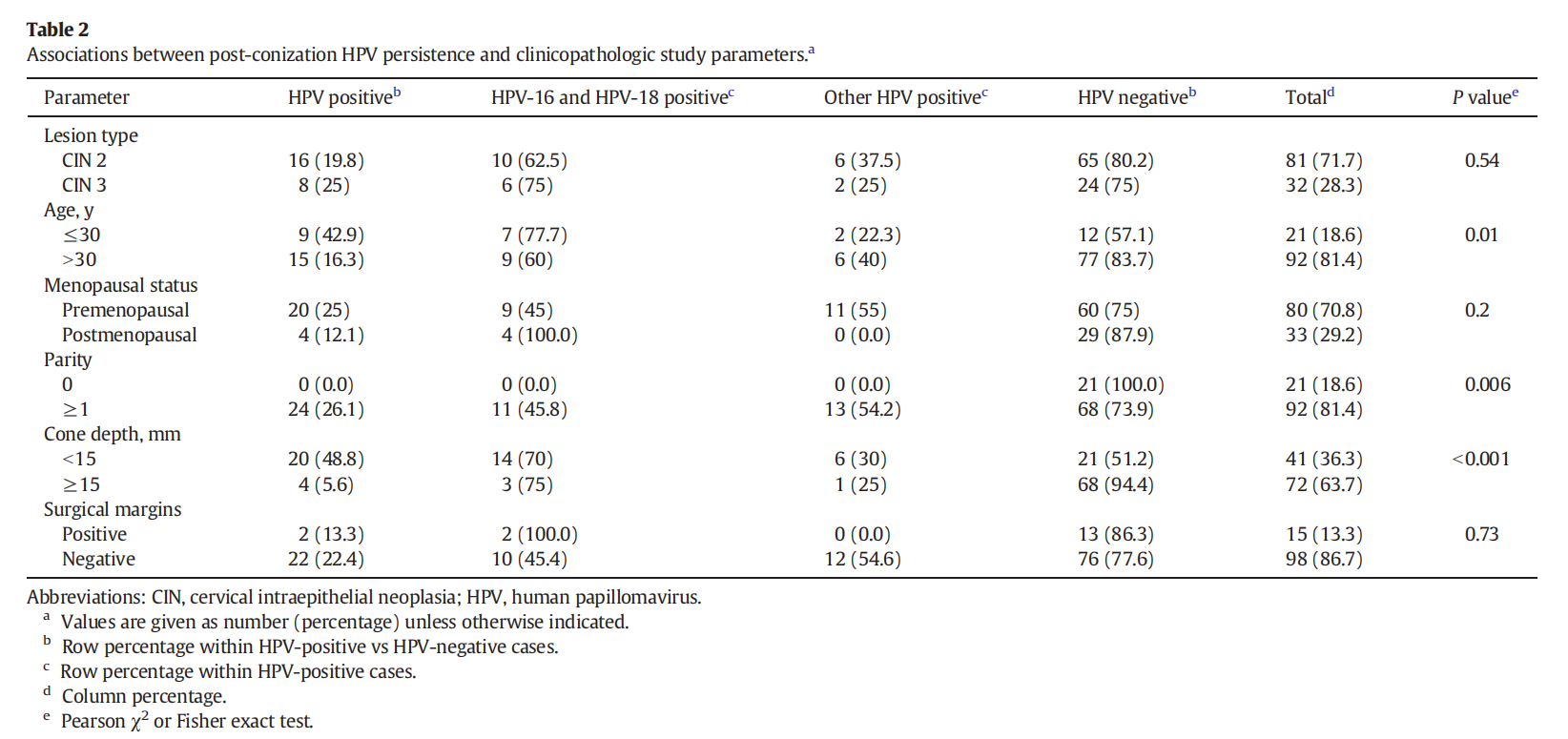


1. Progression of cervical intraepithelial neoplasia grade 2 lesions among Japanese women harboring different genotype categories of high-risk human papillomaviruses


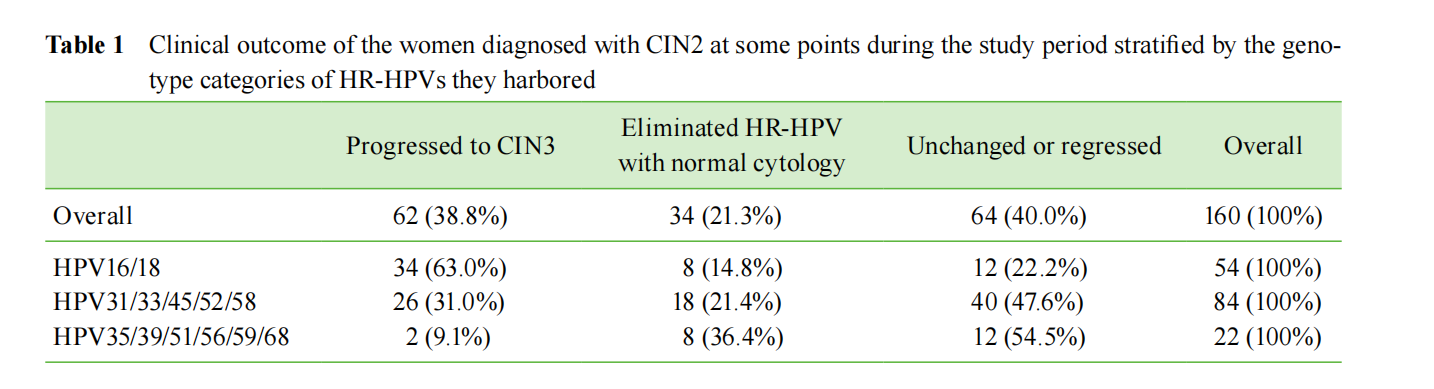


1. The association of pre-conization high-risk HPV load and the persistence of HPV infection and persistence/recurrence of cervical intraepithelial neoplasia after conization


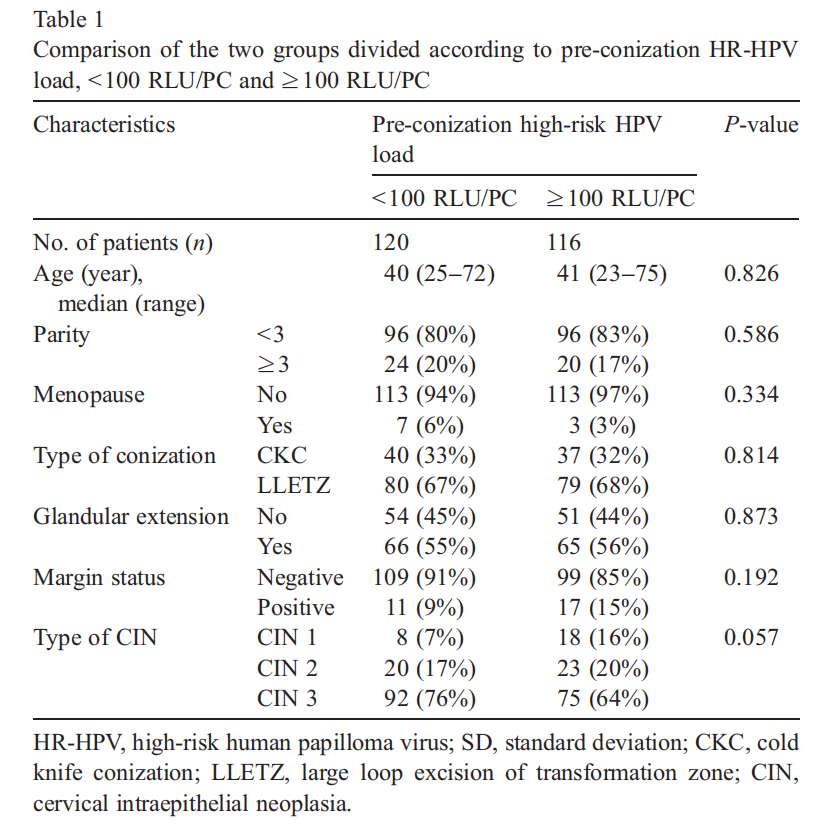


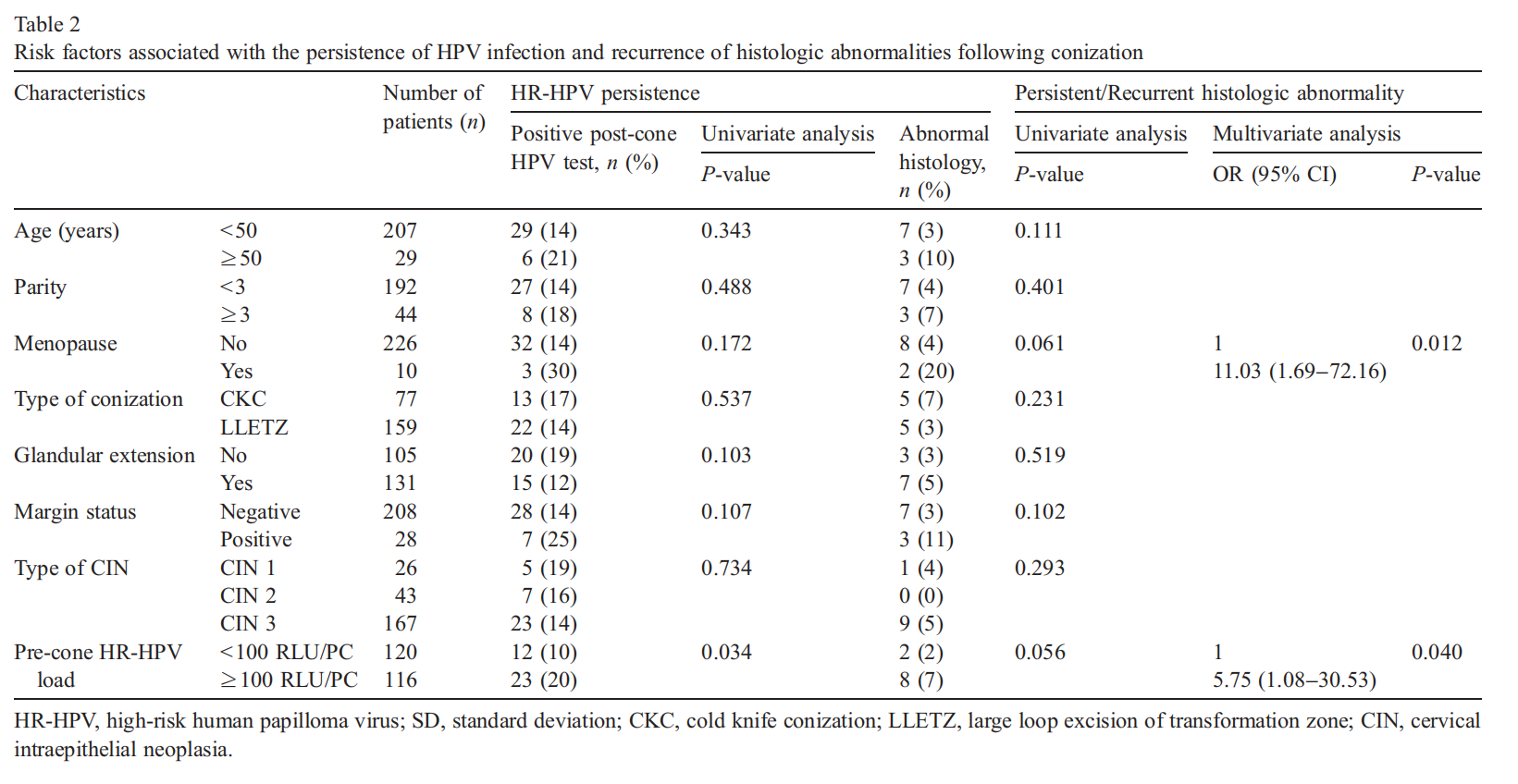


1. Factors associated with HPV persistence after treatment for high-grade cervical intra-epithelial neoplasia with large loop excision of the transformation zone (LLETZ)


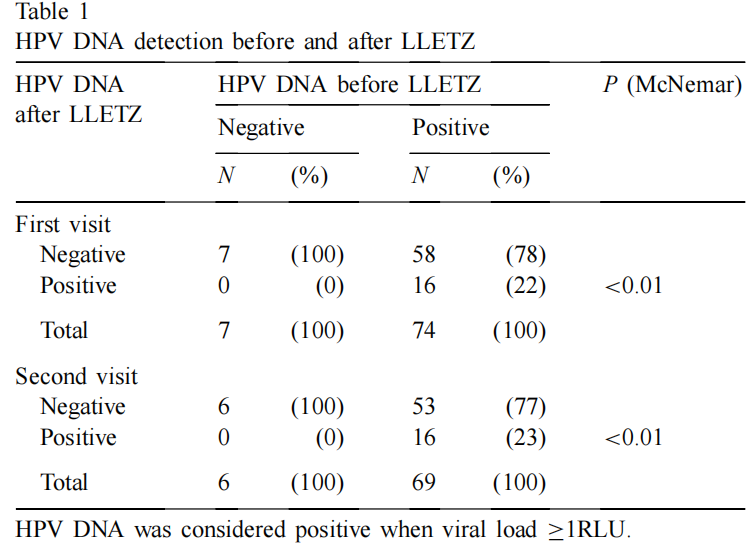

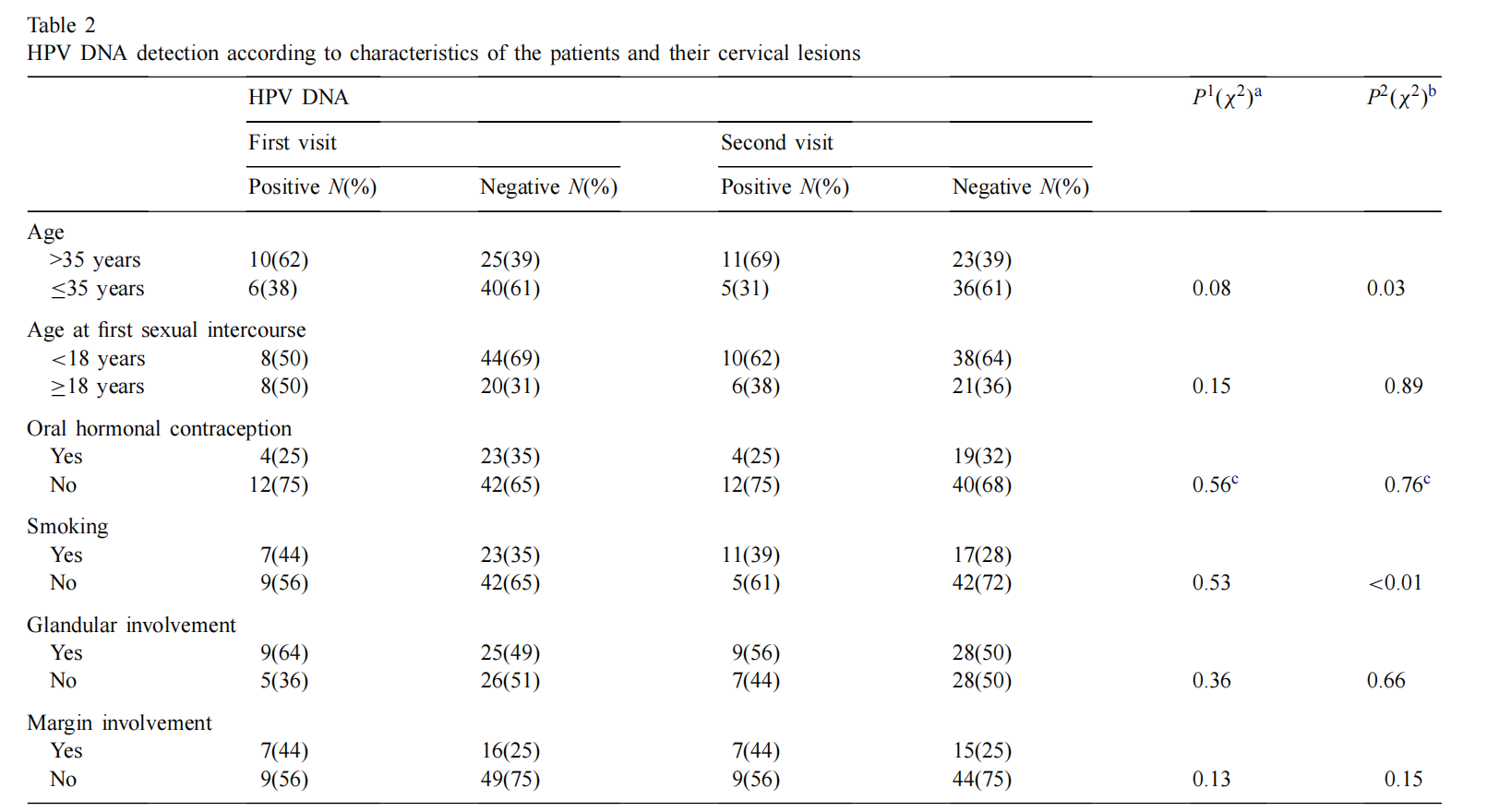


1. Human papillomavirus type-specific persistence and reappearance after successful conization in patients with cervical intraepithelial neoplasia


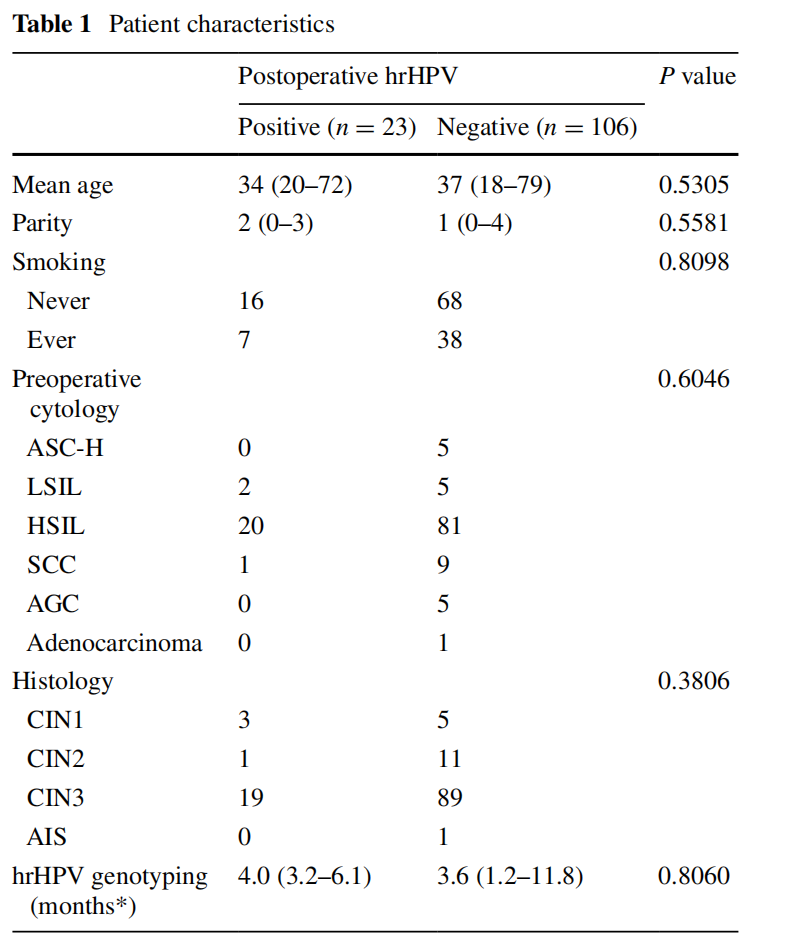


1. Age, margin status, high-risk human papillomavirus and cytology independently predict recurrent high-grade cervical intraepithelial neoplasia up to 6 years after treatment


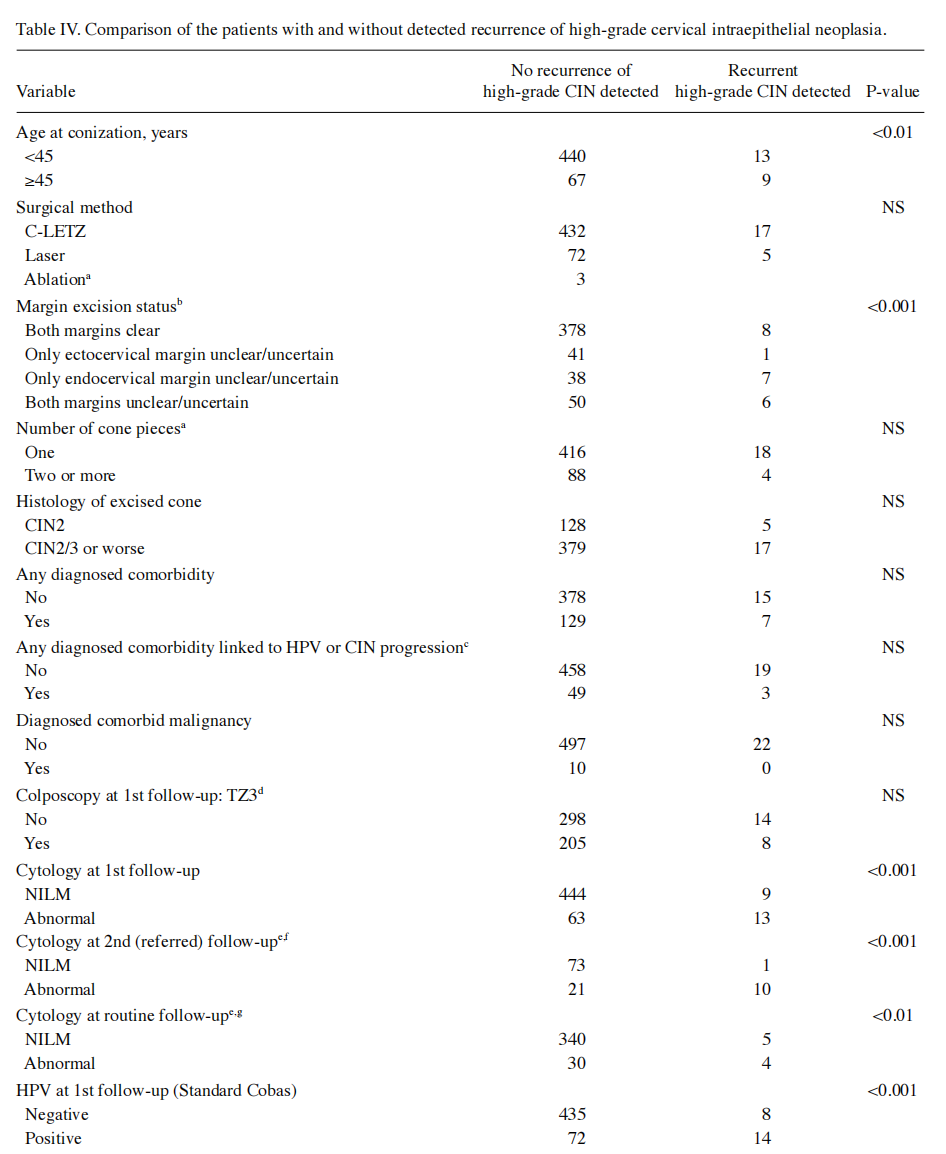


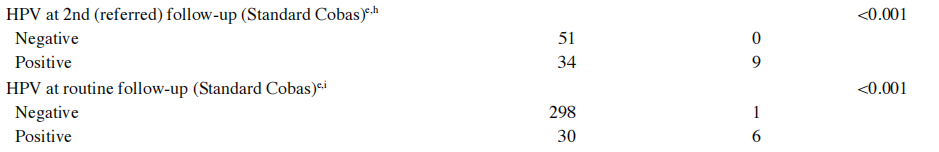


1. Clinical Significance of the Interaction between Human Papillomavirus (HPV) Type 16 and Other High-Risk Human Papillomaviruses in Women with Cervical Intraepithelial Neoplasia (CIN) and Invasive Cervical Cancer


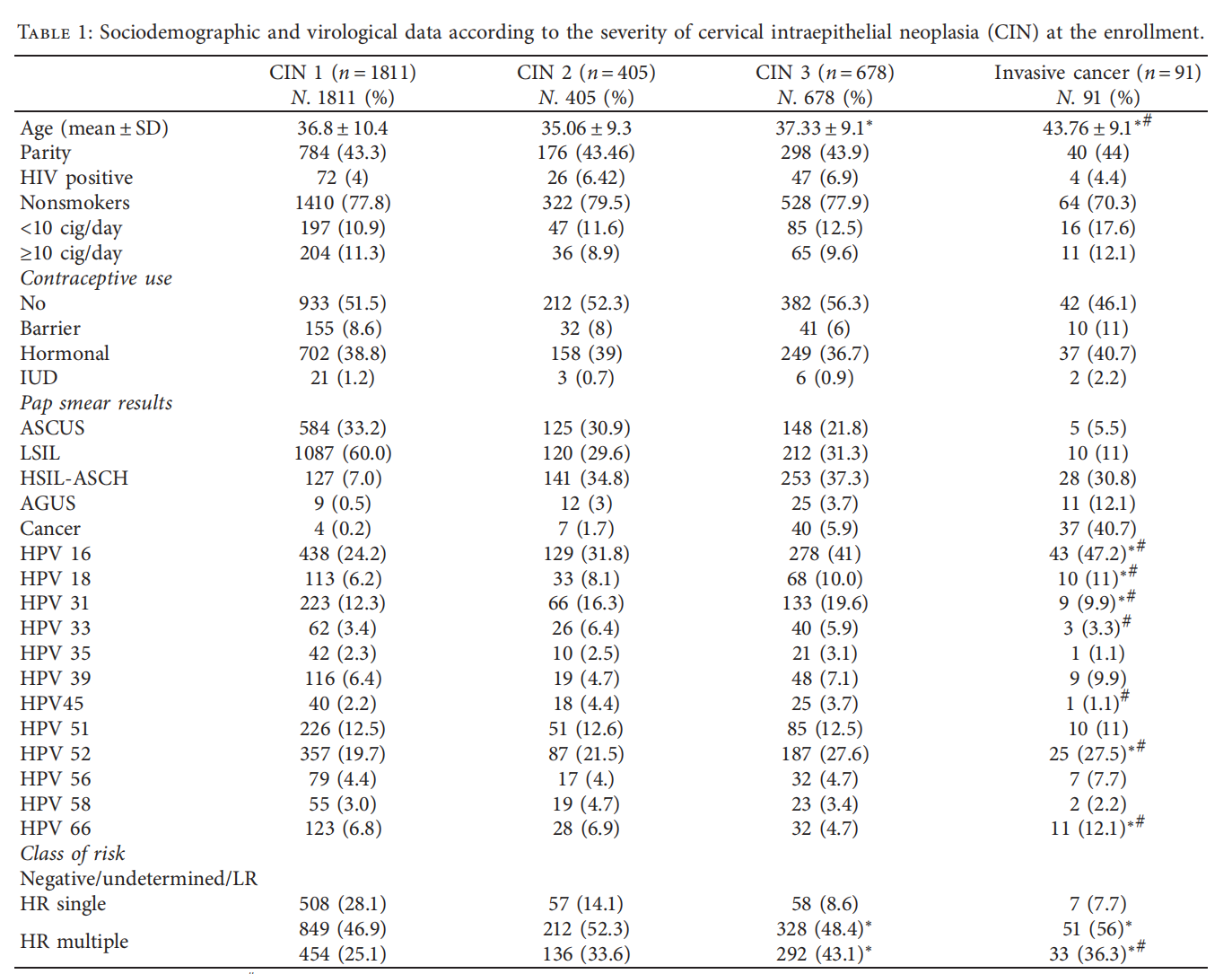

Supplement: Supplementary file 1 — Additional file 1. [file 12905_2023_2360_MOESM1_ESM.doc]
